# Supplementary material for: Isolation of neural stem and oligodendrocyte progenitor cells from the brain of live rats
Source: Stem Cell Reports. 2021 Sep 23;16(10):2534–47. doi: 10.1016/j.stemcr.2021.08.015 (PMC8514974; doi:10.1016/j.stemcr.2021.08.015)
Supplement: Document S1. Supplemental experimental procedures and Figures S1–S6 [file mmc1.pdf]

Stem Cell Reports, Volume *16*

Supplemental Information

Isolation of neural stem and oligodendrocyte progenitor cells from the brain of live rats

Freyja McClenahan, Christina Dimitriou, Christos Koutsakis, Dimitrios Dimitrakopoulos, Asterios Arampatzis, Paraskevi Kakouri, Michaela Kourla, Sofia Oikonomou, Evangelia Andreopoulou, Melina Patsonis, Danai-Kassandra Meri, Rana-Tahir Rasool, Robin JM. Franklin, and Ilias Kazanis

Supplemental Figures and Legends; Legends to Supplemental Movies

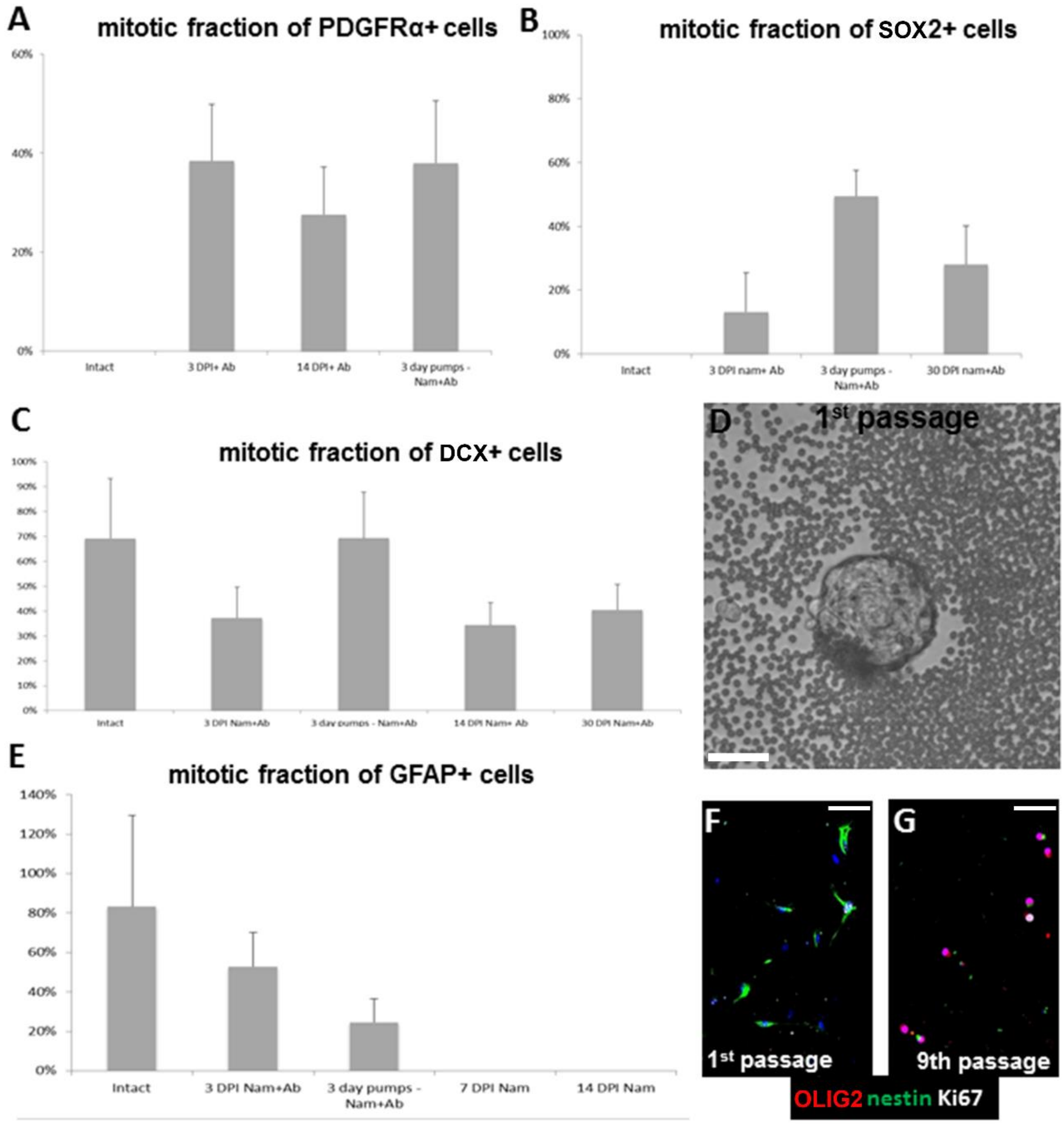

Suppl. Figure 1 (Related to Fig2)

Mitotic profile of cells in liquid biopsies

(A-C,E) Graphs showing the percentage of PCNA/PDGFRα (in A), PCNA/SOX2 (in B), PCNA/DCX (in C) and PCNA/GFAP (in E) double-positive cells collected per liquid biopsy of CSF at different time-points after the “basic release cocktail” (500mU neuraminidase, 1µg β1-integrin blocking Ab) injection as well as after 3 days of infusion via minipump. [error bars: SEM, statistical analysis using one-way ANOVA; n=3-6 animals per experimental group]. (D) Characteristic brightfield image of a primary neurosphere growing in the presence of erythrocytes. (F-G) Images of cells immunostained for OLIG2, nestin and Ki67, showing increased presence of oligodendroglial lineage cells in high passages. [scale bars: 50µm in D, 25µm in F,G]

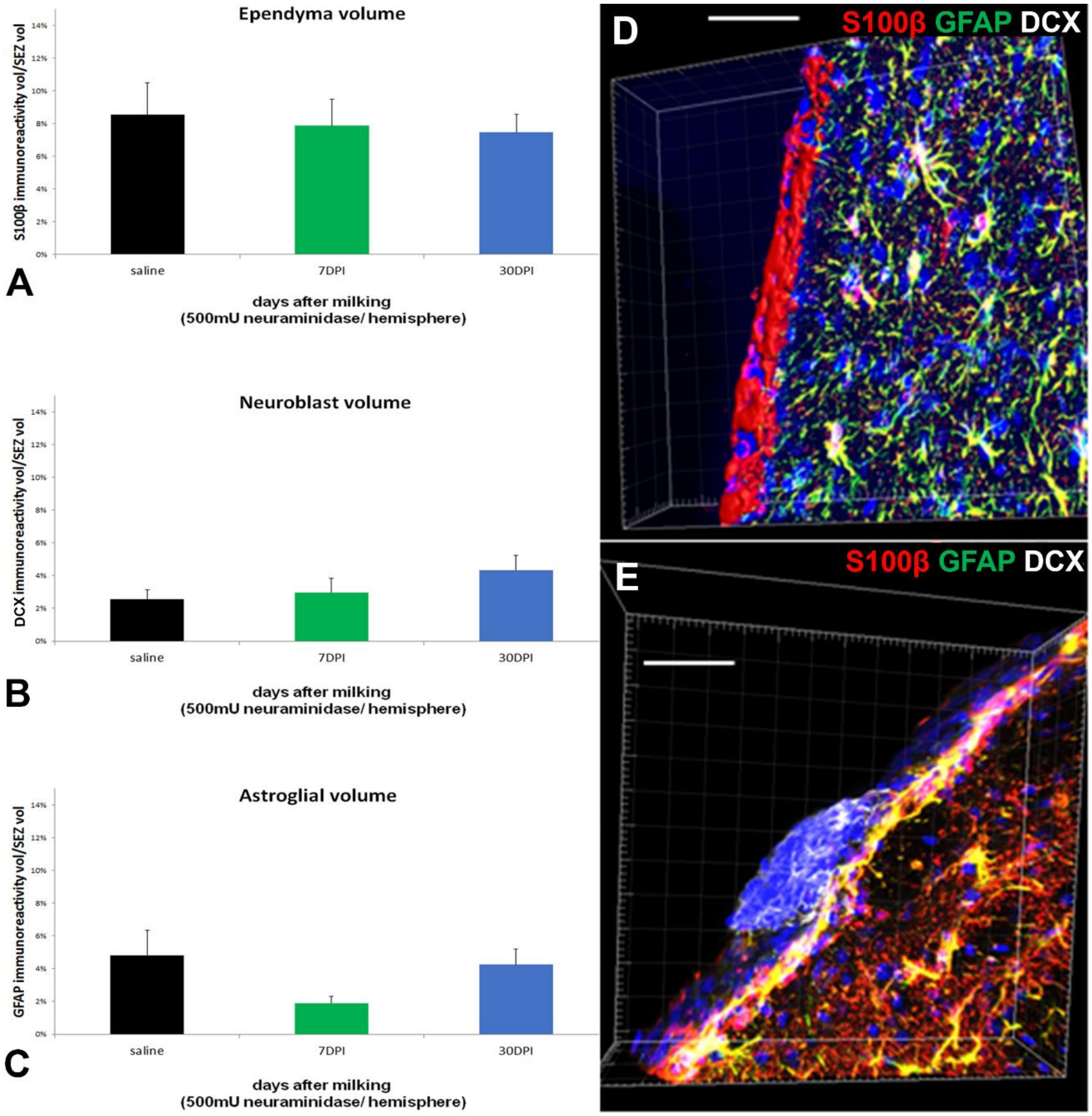

Suppl. Figure 2 (Related to Fig4 and Fig5)

Volumetric analysis of the post-milking SEZ

(A,C) Graphs showing the volume of the ependyma, of neuroblasts and of astrocytes (as volume fraction of S100β, DCX and GFAP immunopositive cell-somata and processes, respectively, per total SEZ volume) at different time-points after the injection of the release cocktail that contained 0.5U neuraminidase per injection (D,E). Representative Imaris-produced images of the saline-injected (in D) and of the 7DPI (in E) SEZ area after immunostaining for S100β, GFAP and DCX. Note a cluster of neuroblasts at the ventricular surface after milking of the SEZ in (E). [error bars: SEM; scale bar:150μm; one-way ANOVA, n=3 animals per experimental group]

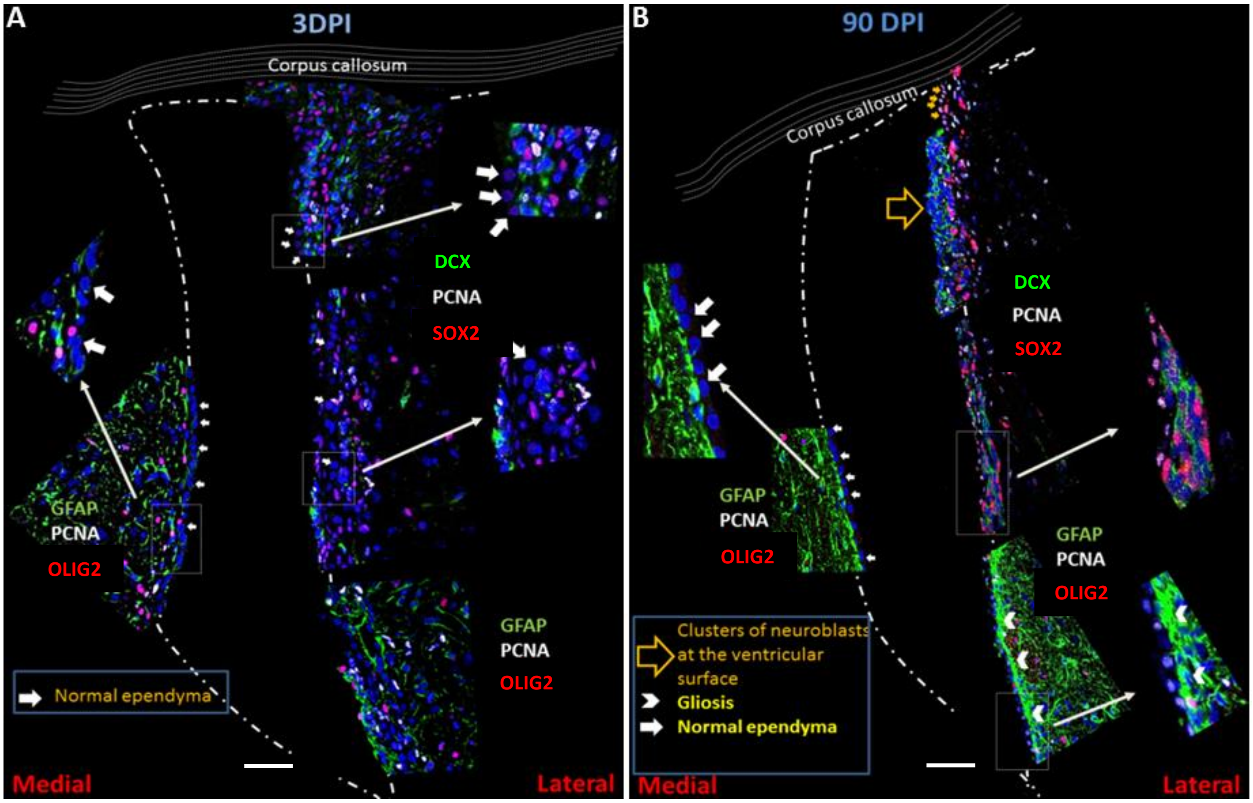

Suppl. Figure 3 (Related to Fig4)

Histological analysis of the post-milking SEZ
(A,B) Collage of images taken from the SEZs of rats at 3 days (in A) and 90 days (in B) after the injection of the "0.5U neuraminidase + blocking Ab" release cocktail. The collages are constructed with images taken from different sections of the same animals, after immunostaining for DCX/PCNA/SOX2 and GFAP/PCNA/OLIG2. Note areas of normal ependyma (indicated by white arrows), especially at the medial (non-neurogenic) wall of the lateral ventricles, as well as the existence of clusters of neuroblasts on the ventricular surface at 3 months post-injection. Also, note the emergence of areas of gliosis 3 months post-milking [scale bar: 50μm].

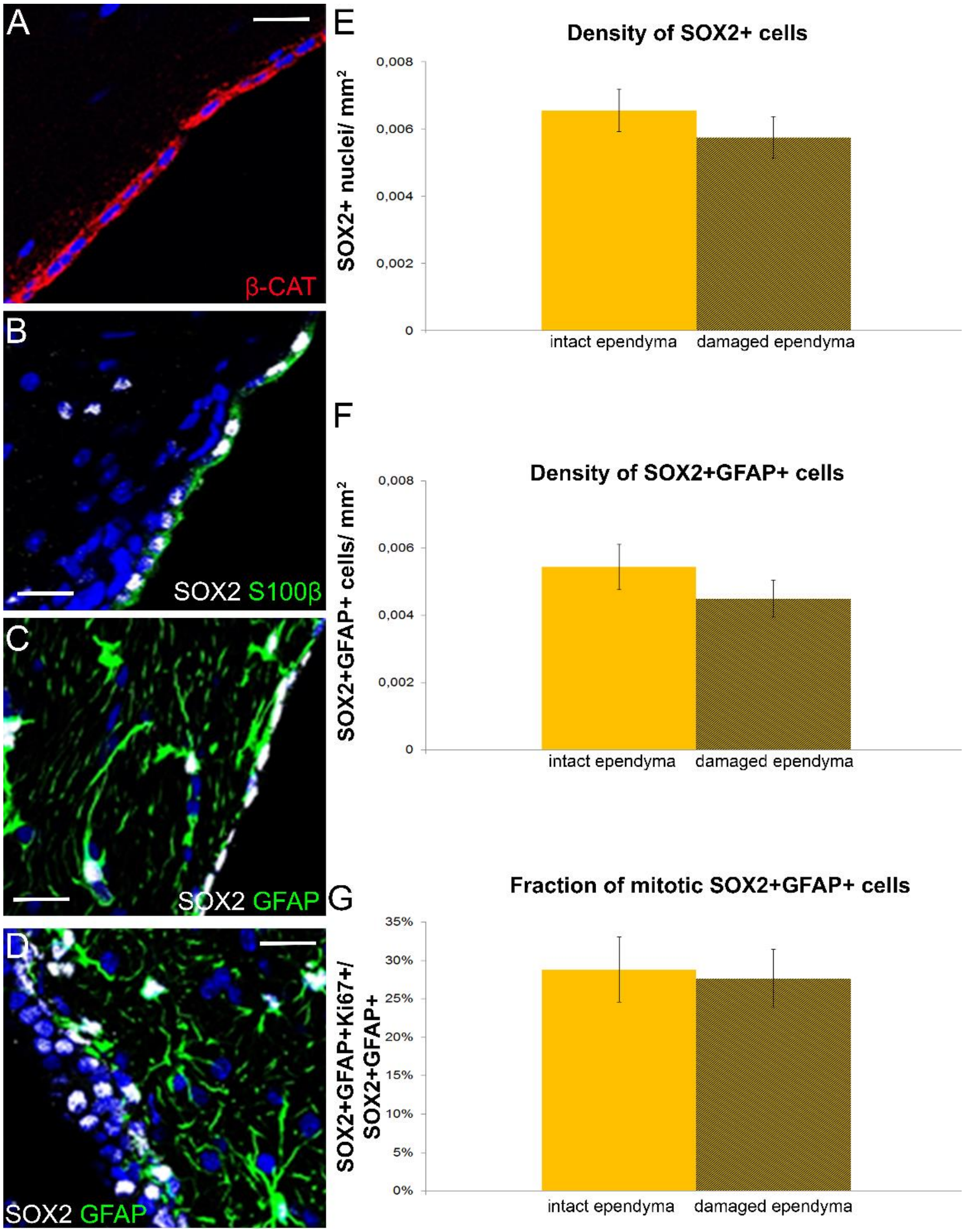

Suppl. Figure 4 (Related to Fig5)

Histological analysis of the SEZ in respect to ependymal damage at 8 months post-injection

(A-D) Details of the SEZ 8 months (240DPI) after the injection of the "+FGF2 release cocktail" and after immunostaining for β-catenin (in red, in A, to mark ependymal cells), SOX2 (in white in B-D), S100β (in green, in B, to mark ependymal cells) and GFAP (in green, in C-D). Images were taken from the same animal, with those in A-C, depicting SEZ domains with intact ependymal (note the smooth ependymal monolayer) and that in D depicting a detail from an area of damaged (note the lack of a continuous SOX2+ monolayer). (F-G) Graphs showing the average percentages of total SOX2+ ependymal and NSPC, of SOX2/GFAP double positive, activated astrocytes and of mitotic, activated astrocytes, in areas adjacent to intact and damaged ependyma. [scale bar: 20μm; error bars: SEMs, statistical analysis paired t-test; n=4 animals]

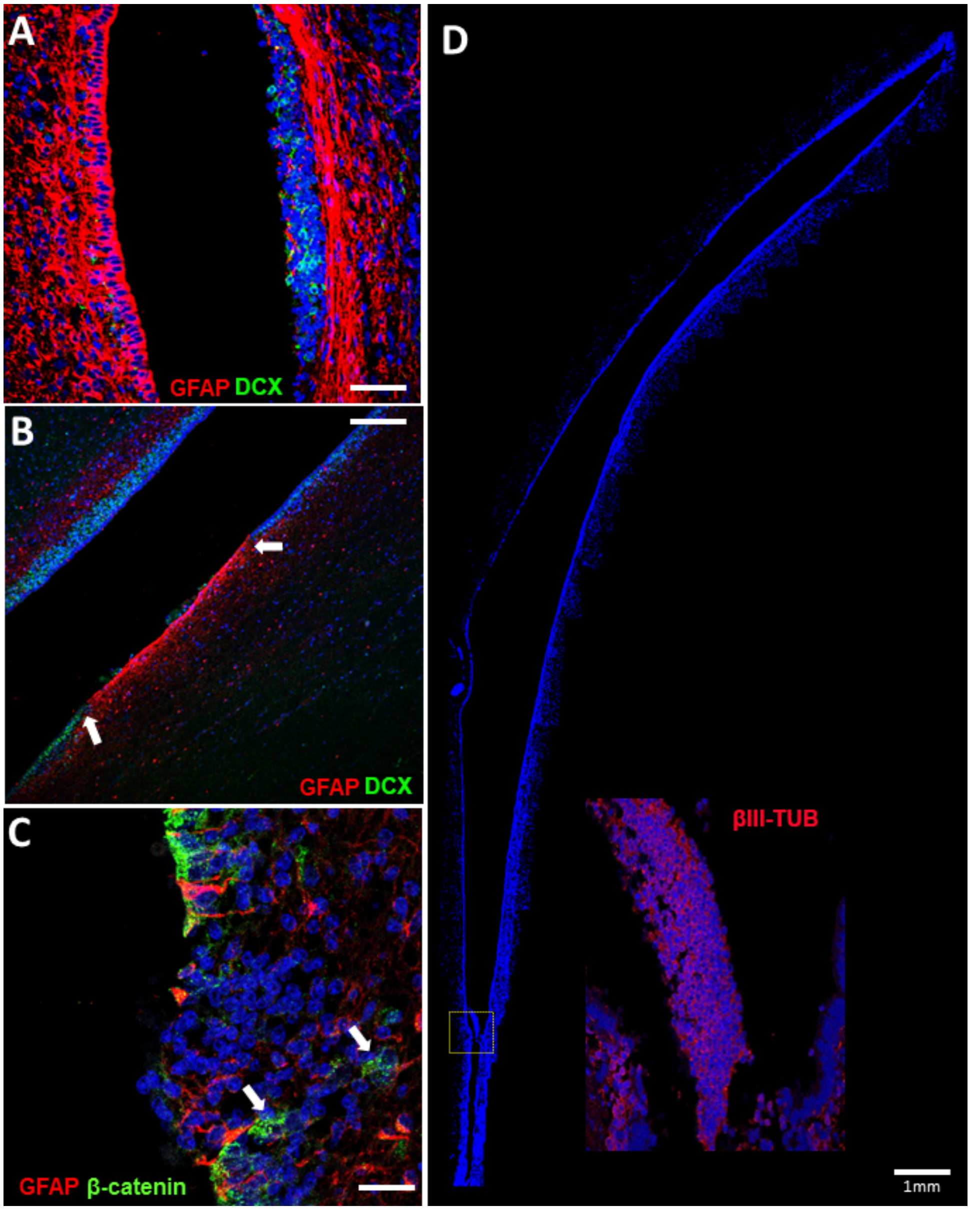

Suppl. Figure 5 (Related to Fig6)

Detection of ependymal disruption, gliosis and neuroblast clustering in the human infant SEZ

Images of the SEZ in tissue taken from newborn infants and immunostained for several markers reveal areas of gliosis and overlaying clusters of neuroblasts (in A and B; gliotic scar indicated by arrows in B) and of groups of neuroblasts flowing in the CSF (D; the boxed area is shown in magnification in the inset). Immunostaining for β-catenin helps visualize the disruption of the ependymal layer (in C) with some ependymal cells surviving deeper in the tissue (indicated with arrows in C). [scale bars: 50µm in A,C; Image D is a collage of multiple photos of Dapi staining]

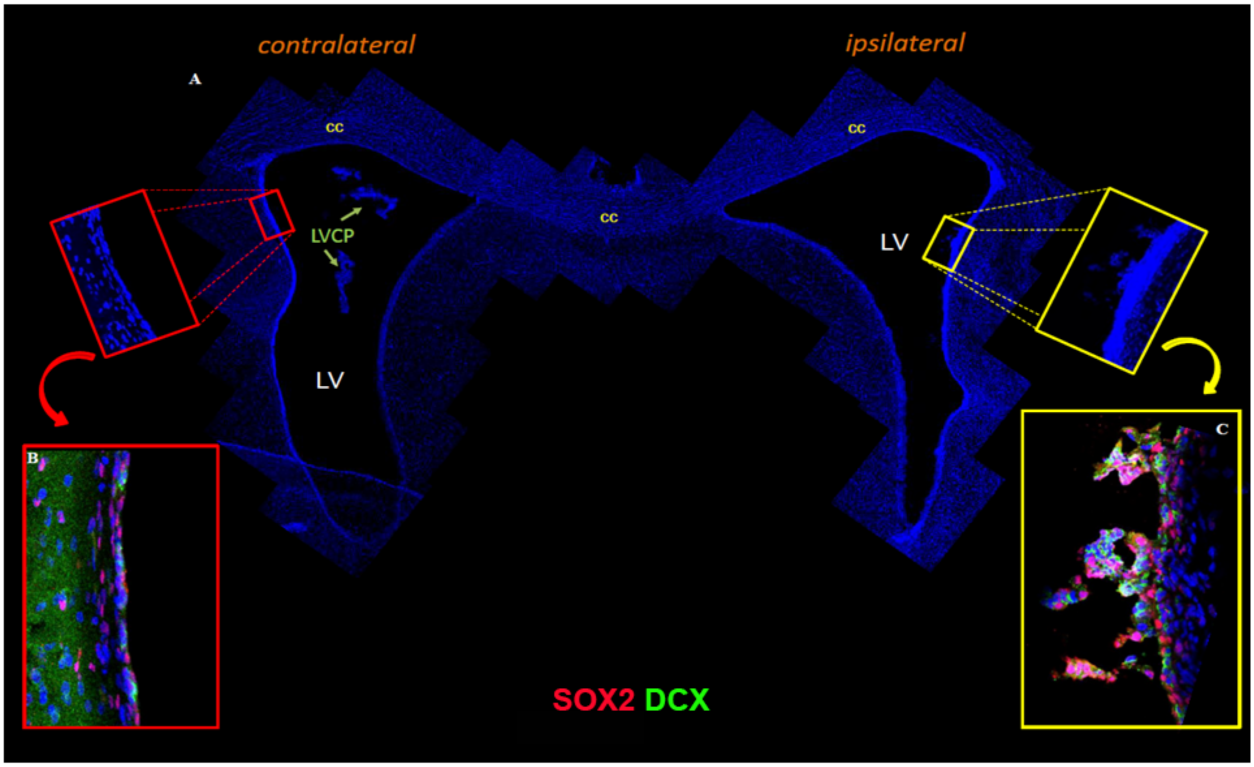

Suppl. Figure 6 (Related to Fig6)

Detection of ependymal disruption and neuroblast clustering in the SEZ post hypoxia/ ischaemia

Collage of images showing the lateral ventricles of a rat 4 weeks after hypoxic/ischaemic injury (60min of Middle Cerebral Artery Occlusion/ MCAO) and after immunostaining for DCX and SOX2. Ependymal disruption accompanied by the flow of SOX2+/DCX+ cells in the ventricular space is observed at the affected hemisphere (at the right).[LV: Lateral Ventricle; LVCP: Lateral Ventricle Choroid Plexus; cc: corpus callosum

Suppl. Movie 1 (Related to Fig1,3)
Detail of the intact SEZ ventricular surface
Movie generated by confocal-microscopy-derived stack of images, taken from rat tissue after immunostaining for GFAP. Note the GFAP-negative lining of the ventricular wall (made of ependymal cells).

Suppl. Movie 2 (Related to Fig1,3)
Detail of the post-milking SEZ ventricular surface
Movie generated by confocal-microscopy-derived stack of images, taken from rat tissue 90 days post-milking, after immunostaining for GFAP. Note the existence of only sporadic GFAP-negative cells on the ventricular wall (surviving ependymal cells) and the gliotic scar that is forming at the ventricular wall.

Suppl. Movie 3 (Related to all Figures)
Summary animation of the "milking" method.

Supplementary Experimental Procedures (Related to Experimental Procedures)

Tissue processing and immunohistochemistry

Animals were culled by transcardial infusion of 4% paraformaldehyde (PFA), tissue was post-fixed overnight in 2% PFA (at 4°C) and was cryo-preserved in 30% sucrose (in Phosphate Buffer Saline/PBS) for 48h at 4°C before freezing at -50°C (for cryostat) or storage in anti-freezing solution at -20°C (for vibratome). Sections were cut with a Leica cryostat (12 mm thick), or a Leica vibratome (70μm) and were processed for immunohistochemistry using the primary antibodies listed below. Whole mount preparations of the SEZ were performed as previously described[2].

Immunofluorescence stainings were performed using standard protocols, that included incubation with blocking buffer (3%BSA, 0.1% Triton x-100 from Sigma, UK, in PBS) and, depending on the requirement, antigen retrieval (15min boiling in 10mM citrate buffer, pH=6.0). Human tissue was provided in the form of thin paraffin sections; thus, they underwent a deparaffinization/rehydration process before further manipulation. Primary antibody incubation (in blocking buffer) was performed for one or two overnights at 4°C and incubation with secondary antibodies (in PBS with Dapi for nuclear staining) for 2h at room temperature. Slides were covered with coverslips mounted with mowiol. Images were acquired using Leica SP5 and SP6 confocal microscopes and were processed using ImageJ (NIH, USA) and LasX (Leica) software.

Antibodies and Lectin

Rabbit anti-OLIG2 (1/200, Millipore, AB9610) and rabbit anti-PDGFRα (1/200, Abcam, 1/200, ab51875) were used to identify OPCs. Rabbit, goat, chicken or mouse anti-GFAP (Dako, 1/500, Z0334; Abcam, 1/500, ab53554; Abcam, 1/500, ab4674; Sigma, 1/500, G3893) and rabbit anti-DoublecortinCX (Abcam, 1/500, ab18723) or mouse anti-TUBULIN βIII (Sigma, 1/500, T8578) were used for astrocytes and neuroblasts respectively. To assess progenitor identityidentity, we used a monoclonal anti-NESTIN (1/200, Abcam, ab6142) and a goat anti-SOX2 antibody (Santa Cruz, 1/200, sc-17320). Proliferating cells were identified using a mouse anti-PCNA (Abcam, 1/500, Ab29), a rabbit anti-Ki67 (Abcam, 1/500, ab15580), or a rabbit anti-PH3 (Abcam, 1/500, ab80612) antibody. Ependymal cells were identified using a mouse anti-S100β (Sigma, 1/200, S2532), a rabbit anti-acetylated α-TUBULIN (Sigma, 1/500, T6793) and a rabbit anti-β-CATENIN (Abcam, 1/500, ab16051). Transit amplifying progenitors were marked with an anti-Ascl1 (MASH-1) mouse monoclonal antibody (1/100, Clone 24B72D11.1, (RUO) BD Bioscience, 556604). The

appropriate secondary antibodies were used, purchased from Thermo Fisher (Molecular probes, Alexa conjugated 488, 568 and 647).

Neuraminidase-targeted sialic acid residues (Sialic acid α(2,3) galactose) were identified by binding with Maackia amurensis lectin (MAA, BA-7801-2, EY Laboratories Inc.) that was biotin-conjugated and was subsequently visualized using Streptavidin conjugated with Alexa 594 (Thermo Fisher).

Detailed milcking protocol

A. "Release cocktail" composition (prepared at the day of the procedure and kept on ice. Quantities given per 2µl to be injected i.cv in each lateral ventricle):

1. 500mU Neuraminidase from Clostridium perfringens (*Clostridium welchii*) (#N2876,Sigma-Aldrich) [kept as a 1U/µl stock diluted in sterile water at -20°C]**[0.5µl]**
2. 1µg β1-integrin blocking antibody (#555002, BD Biosciences) [purified NA/LE Hamster Anti-Rat CD29 Clone Ha2/5, 1mg/ml, kept at 4°C]**[1µl]**
3. 0.5µg Fibroblast Growth Factor (#100-18B, Peprotech)[Recombinant Human FGF-basic (154 a.a.), kept as a 1µg/µl stock diluted in sterile water at -20°C]**[0.5µl]**

Note: prepare an additional 1µl per intended injection

B. Injection of "release cocktail":

Note: surgical procedures were performed under general anaesthesia induced by inhaled isofluorane (2.5% for induction and 2% for maintenance), but can be successfully performed under injectable anaesthesia (e.g. ketamine/ xylazine). On average the procedure lasted for 30min and analgesia (Vetergesic; Buprenorphine 0.3mg/ml as buprenorphine hydrochloride 0.324 mg/ml) was given i.p. upon induction of anaesthesia.

4. Standard procedures for mounting the animal on the stereotaxic frame are followed. An incision of the head's skin along the middle line is performed, followed by meticulous clearing of the skull, identification of bregma and the drilling of 1mm burr holes, using a dental drill, at the following co-ordinates (from bregma): Anterioposterior axis +0.5mm, Lateral axis ±1.4mm).
5. A loaded 10µl Hamilton syringe is fixed on the stereotaxic device and the needle (preferably blunt or conical edge) is brought in contact with the dura and then inserted 3.5mm deep.

6. The "release cocktail" is infused at a rate of 1μl/min and then the needle is left for another 2min before being retrieved. The procedure is repeated at the other hemisphere.
7. Incisions are sutured and the animal is transferred to the post-operation monitoring area.

C. CSF liquid biopsy:

Note: surgical procedures were performed under general anaesthesia induced by inhaled isofluorane (2.5% for induction and 2% for maintenance), but can be successfully performed under injectable anaesthesia (e.g. ketamine/ xylazine). On average the procedure lasted for 10min; analgesia was given at the induction of anaesthesia.

8. The animal is mounted on the stereotaxic frame using only the ear bars; thus, allowing free forward/backward rotation of the head.
9. The head is stabilized at a downward 40° angle so that a good extension of the back of the neck can be achieved; the fur is shaved and the skin is cleaned.
10. A depressible surface with the appearance of a rhomb between the occipital protuberance and the spine of the atlas can be identified using the tip of a finger (Consiglio and Lucion, 2000; Pegg et al., 2010).
11. A 1ml insulin-like syringe is fixed on the stereotaxic frame and a 27G needle is attached on it (syringes with compact (non-detachable) needles are preferable as suction is more efficient and there is less dead-volume). The needle is lowered almost at contact point to the skin at the center of the identified rhomb. After the syringe is lowered further through the skin layers, small suction is applied to create negative pressure. The syringe is lowered at very small steps until CSF starts to appear due to the negative pressure.
12. The syringe is stabilized at this point and further suction is applied to enable more CSF flow. The needle can be lowered or elevated slightly. The suction of CSF, at a rate of 40μl/ min, will produce a clear from blood sample of up to 120μl. The syringe must be removed slowly and the rat should be supported by an i.p. injection of 1ml normal serum.
13. The liquid biopsy is mixed with 400μl of NSPC medium [DMEM (Thermo Fisher), B27 supplement (2% v.v) (Thermo Fisher), 20ng/ml FGF2 (Peprotech) and 20ng/ml EGF (Peprotech)] and is kept at 4°C until further use. (*Note: all liquid biopsies were processed the latest after 4h)*
14. The animal is transferred to the post-operation monitoring area.

References

Consiglio, A.R., and Lucion, A.B. (2000). Technique for collecting cerebrospinal fluid in the cisterna magna of non-anesthetized rats. Brain Res Brain Res Protoc *5*, 109-114.

Pegg, C.C., He, C., Stroink, A.R., Kattner, K.A., and Wang, C.X. (2010). Technique for collection of cerebrospinal fluid from the cisterna magna in rat. J Neurosci Methods *187*, 8-12.
