# Supplementary material for: Isolation of neural stem and oligodendrocyte progenitor cells from the brain of live rats
Source: Stem Cell Reports. 2021 Sep 23;16(10):2534–47. doi: 10.1016/j.stemcr.2021.08.015 (PMC8514974; doi:10.1016/j.stemcr.2021.08.015)
Supplement: Document S2. Article plus supplemental information [file mmc5.pdf]

Stem Cell Reports
Article

OPEN ACCESS

Isolation of neural stem and oligodendrocyte progenitor cells from the brain of live rats

Freyja McClenahan,[1,3] Christina Dimitriou,[2,3] Christos Koutsakis,[2] Dimitrios Dimitrakopoulos,[2] Asterios Arampatzis,[1] Paraskevi Kakouri,[2] Michaela Kourla,[2] Sofia Oikonomou,[2] Evangelia Andreopoulou,[2] Melina Patsonis,[2] Danai-Kassandra Meri,[2] Rana-Tahir Rasool,[1] Robin JM. Franklin,[1] and Ilias Kazanis[1,2,*]

[1]Wellcome-MRC Cambridge Stem Cell Institute, University of Cambridge, CB2 0AW Cambridge, UK
[2]Lab of Developmental Biology, Department of Biology, University of Patras, 26504 Patras, Greece
[3]These authors contributed equally
*Correspondence: ikazanis@upatras.gr or ik255@cam.ac.uk (I.K.)
https://doi.org/10.1016/j.stemcr.2021.08.015

SUMMARY

Postnatal brain neural stem and progenitor cells (NSPCs) cluster in anatomically inaccessible stem cell niches, such as the subependymal zone (SEZ). Here, we describe a method for the isolation of NSPCs from live animals, which we term "milking." The intracerebroventricular injection of a release cocktail, containing neuraminidase, integrin-β1-blocking antibody, and fibroblast growth factor 2, induces the controlled flow of NSPCs in the cerebrospinal fluid, where they are collected via liquid biopsies. Isolated cells retain key *in vivo* self-renewal properties and their cell-type profile reflects the cell composition of their source area, while the function of the niche is sustained even 8 months post-milking. By changing the target area more caudally, we also isolate oligodendrocyte progenitor cells (OPCs) from the corpus callosum. This novel approach for sampling NSPCs and OPCs paves the way for performing longitudinal studies in experimental animals, for more *in vivo* relevant cell culture assays, and for future clinical neuro-regenerative applications.

INTRODUCTION

Tissue-specific stem cells (t-SCs) reside in mature tissues in order to maintain homeostasis and to contribute to regeneration, functions they can perform because they retain the cardinal properties of stem cells: self-renewal and the potential to generate multiple, although tissue-specific, cell types. In addition to cells with broad cell-type generation potential, such as embryonic stem cells (ESCs) and induced pluripotent stem cells (iPSCs) (Shi et al., 2017; Theunissen and Jaenisch, 2014) the isolation of t-SCs can serve not only as an alternative source of stem cells but, importantly, and as the most reliable comparator to how ESCs and iPSCs programmed toward specific tissue fates should behave; thus, remains of high experimental and clinical importance.

Postnatal brain neural stem and progenitor cells (NSPCs) reside in anatomically restricted niches of the postnatal brain, such as the subependymal zone [(SEZ) also known as the subventricular zone] of the lateral walls of the lateral ventricles and the subgranular zone of the dentate gyrus (Obernier and Alvarez-Buylla, 2019). Neural stem cells (NSCs) clustering in the SEZ generate transit-amplifying progenitors that subsequently give rise to progeny of neuronal or oligodendroglial commitment (neuroblasts and oligodendroblasts, respectively) (Kazanis et al., 2017). In the rodent brain, neuroblasts migrate to the olfactory bulbs where they differentiate into a range of functional interneurons (Mouret et al., 2009), while oligodendroblasts migrate to the corpus callosum (Etxeberria et al., 2010; Kazanis et al., 2017). The SEZ niche is confined in few cell layers next to the surface of the ventricle (up to 50 μm deep in the rat brain), characterized by a specialized microenvironment in terms of extracellular matrix (Kazanis et al., 2010; Mercier et al., 2002) and blood vessel architecture (Culver et al., 2013; Shen et al., 2008), with NSCs positioned adjacent to ependymal cells. Due to the scarcity of these cells, especially in the adult human brain (Coletti et al., 2018; Sanai et al., 2011), and their location in inaccessible areas, *in vitro* experimental work aiming at deciphering the properties of endogenous NSC populations has relied on the post-mortem isolation of animal NSPCs (Pastrana et al., 2009) and their clinical use remains challenging, albeit of great importance.

Here, we present a novel method that allows the isolation of SEZ-derived NSPCs, as well as corpus callosum oligodendrocyte progenitor cells (OPCs), via cerebrospinal fluid (CSF) liquid biopsies in live animals. Isolated NSPCs cells present a marker profile identical to that of endogenous NSPCs and changes that occur in the SEZ are reflected in the biopsies. Collected cells retain *in vitro* the characteristic properties of SEZ cells, especially their quiescence and their self-renewing capacity, as has been recently reported based on *in vivo* clonal analysis experiments (Calzolari et al., 2015; Obernier et al., 2018). Since multiple biopsies can be performed in the same animal, we have termed it "milking of the SEZ" and, as it does not compromise the function of the SEZ, this method paves the way to longitudinal experimental analyses.

© 2021 The Author(s).
This is an open access article under the CC BY license (http://creativecommons.org/licenses/by/4.0/).

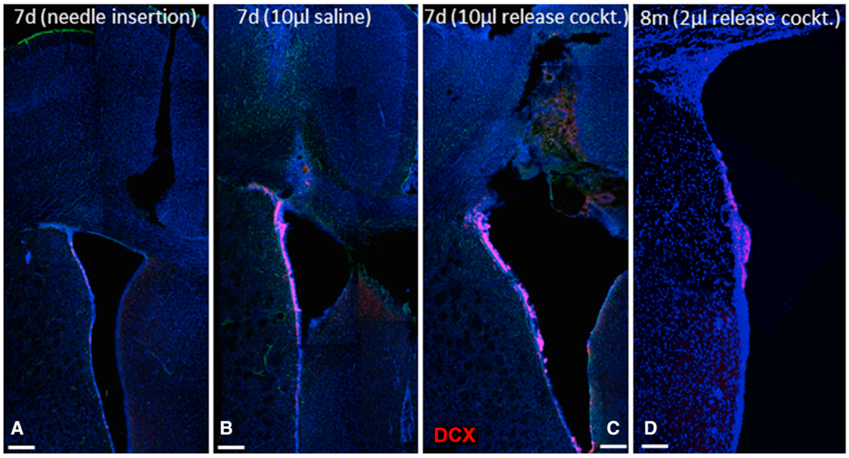

Figure 1. Histological assessment of the effects of i.c.v. injections

Low-magnification images of the middle and dorsal parts of the lateral ventricle after immunostaining for DCX (in red, to mark neuroblasts). The simple i.c.v. insertion of a Hamilton syringe does not disturb the cytoarchitecture of the SEZ in (A), while the i.c.v. injection of 10 µL (infusion rate of 1 µL/min) leads to severe damage of the ventricular wall irrespective of its content; saline in (B) and "release cocktail" in (C). The injection of 2 µL of the release cocktail leads to a controlled compromise of the ventricular wall, observed even at 8 months after surgery in (D). Scale bars, 300 µm (A–C) and 150 µm (D).

RESULTS

Release and collection of NSPCs

NSPCs of the SEZ are positioned adjacent to ependymal cells, with NSCs remaining in direct contact with the CSF via intercalating mono-ciliated processes (Doetsch et al., 1999, 2002). Neuraminidase can induce ventricular wall denudation via cleavage of sialic acid residues on ependymal cells, leading to neuroblast clustering on the ventricular wall (Del Carmen Gomez-Roldan et al., 2008; Luo et al., 2008). We have documented the flow of neuroblasts in the CSF after intracerebroventricular (i.c.v.) injection of an integrin-β1-blocking antibody (Kazanis et al., 2010). Based on these observations, we designed a strategy to compromise the integrity of the lateral ventricle wall, thereby allowing NSPCs to enter the CSF and to be collected via liquid biopsies.

The protocol was developed in rats and includes two major steps. First, NSPCs are "released" via the i.c.v. injection of a "release cocktail" containing neuraminidase, an integrin-β1-blocking antibody, and fibroblast growth factor 2 (FGF2). The cocktail is stereotaxically injected bilaterally (2 µL injected per ventricle) in the lateral ventricles (coordinates: anterioposterior [AP] = −0.3 mm, lateral [L] = ±1.2 mm, depth [D] = 3.5 mm), at an infusion rate of 1 µL/min. The surgery is tolerated well by the animals, with no mortality linked to this procedure. At a second "collection" step, liquid biopsies of CSF were performed from the cisterna magna of anesthetized rats, using 1-mL insulin syringes. The use of a stereotaxic device allows near absolute success in retrieving approximately 100 µL of CSF, without the need for incisions. The liquid biopsy is mixed with NSPC culture medium and can be kept at 4°C until plating. We have successfully repeated up to three successive liquid biopsies in the same animal, with a minimum of 7 days in between, with only transient weight loss and no signs of locomotor or behavioral defects.

Collected cells have the marker profile of NSPCs, form colonies, and show limited self-renewal capacity

Several parameters, such as the volume of injection, the time point of CSF biopsy, and the release cocktail composition, were assessed during the process of refinement of the "milking" protocol. Initial experiments (n = 20) revealed that the injection of more than 3 µL of vehicle (saline) resulted in non-specific uncontrolled mechanical rupture of the ventricular wall (Figure 1). They also showed that the addition of the integrin-β1-blocking antibody increased the number of free-floating neuroblast clusters in the CSF, most probably due to the loosening of cell-cell interactions between neighboring ependymal cells that express high levels of integrin-β1 (Kazanis et al., 2010), as has shown for integrin-β1-expressing ventricular zone progenitors in the developing cortex (Loulier et al., 2009). Next, we assessed cell yields at various days post-injection (dpi), ranging from 3 days (minimum time to allow full post-surgery recovery) up to 30 days, with the addition of an alternative setting in which a cannula attached to an osmotic minipump filled with the release cocktail was implanted i.c.v. and the liquid biopsy was performed after 3 days of infusion. The average cell yield was similar at the different collection time points (3, 7, 14, and 30 dpi) (300 ± 45 cells per biopsy), albeit they were all more productive compared with collections post-saline injection (24 ± 12 cells per biopsy) (Figure 1A). The immunocytochemical cell-type profile of collected cells (GFAP+ astrocytes, DCX+ neuroblasts, PDGFRα+ oligodendrocyte progenitors, SOX2+ and NESTIN+ NSPCs, total PCNA+ proliferating cells, and proliferating fractions of different cell types) was similar, irrespective of the milking protocol (saline, injection, osmotic minipump delivery) with the exception of significantly less PDGFRα+ cells collected at the 14 dpi biopsies and the absence of double GFAP+ PCNA+ cells within collections at 7 and 14 dpi

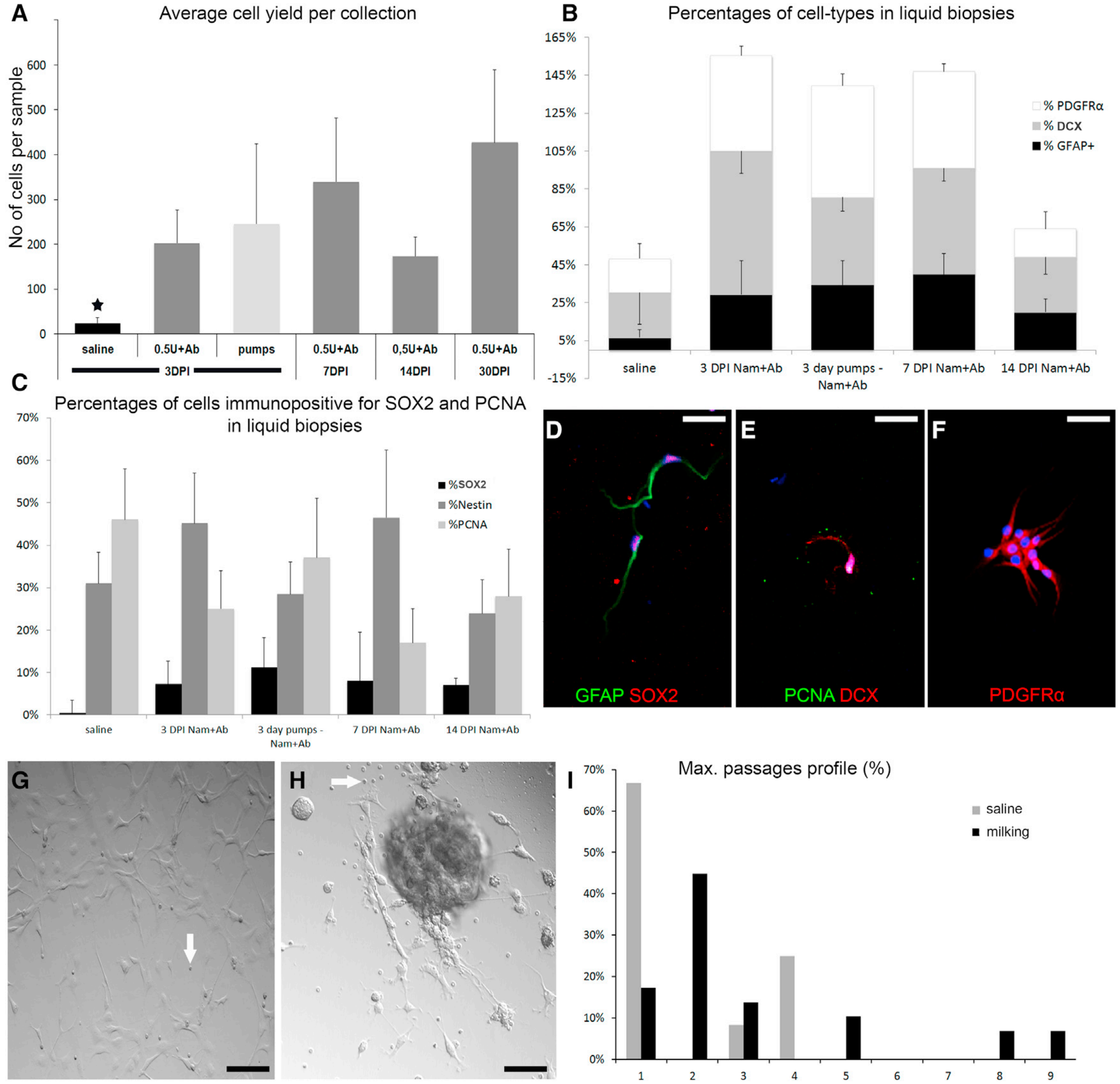

Figure 2. Validation of cell yield and profile in liquid biopsy samples

(A) Graph showing the average number of cells collected per "positive" liquid biopsy of CSF at different time points after the release cocktail injection as well as after 3 days of infusion via minipump (*$p < 0.001$, one-way ANOVA, $n = 5–7$ animals per experimental group).

(B and C) Graphs showing the cell-type profile of cells collected via liquid biopsy of CSF at different time points after the release cocktail injection, as well as after 3 days of infusion via minipump ($n = 5–7$ independent samples per group).

(D–F) High-magnification images of cells collected via liquid biopsy of CSF after the injection of the release cocktail (0.5 mU Nam + Ab) and immunostained for different markers.

(G) Image of a cell colony 10 days after the initial plating of the liquid biopsy sample. Cells show a typical NSPC morphology.

(H) Image of a neurosphere 15 days after the initial plating of the liquid biopsy sample. The optical plane is at the surface of the coverslip and shows adherent cells with typical NSPC morphology, some being connected with the overlaying neurosphere via cellular processes, as well as a few erythrocytes.

(I) Graph showing the maximum number of passages obtained per liquid biopsy sample from saline-injected animals (gray bars, total of 12 samples), or after milking (black bars, total of 29 samples, release cocktail of 0.5 U Nam + Ab). White arrows, erythrocytes; DCX, neuroblasts; GFAP, astrocytes; PCNA, proliferating cells; PDGFRα, oligodendroblasts; SOX2, neural progenitors. Error bars: SEM. Scale bars, 20 μm. See also Figure S1

(Figures 2B–2F and S1). All isolated GFAP+ cells, irrespective of PCNA expression, were immunopositive for SOX2. Thus, liquid biopsies at 3 dpi were chosen as the preferred procedure due to being the shortest collection time point that also provided samples rich in potential NSCs (GFAP/PCNA++) and OPCs (PDGFRα+).

Liquid biopsies were collected with 100% efficiency from all animals (after a short training period, volumes can range between 100 and 120 μL). Samples were kept on ice and plated, the latest after 4 h, on poly-D-lysine-coated plastic or glass surfaces. During the initial pilot experiments, all cells were counted within the first 24 h after plating and a dichotomy was observed, with biopsies producing less than 10 clearly identifiable cells never showing any sign of cell proliferation within the following 15 days; thus, these were subsequently routinely excluded from the rest of the study. Samples with more than 10 cells were called "positive." We performed 125 biopsies after the injection of different release cocktails and 35 from saline-injected rats. The efficiency of obtaining positive biopsies was 83.3% after release and 32.1% after saline ($p < 0.001$, using t test analysis). Nevertheless, in 42.31% of post-release cocktail and 27.78% of post-saline animals, positive biopsy cells did not show signs of colony formation (either adherent or as neurospheres) within 30 days of maintenance. These samples were called "0 passage" cultures. The rest of the biopsies resulted in colony growth, mainly in the form of well-shaped neurospheres attached on the surface, but also as adherent colonies (Figures 2G and 2H). It should be noted that colony formation was not inhibited by the presence of erythrocytes in the biopsies (Figures 2G and S1D). When neurospheres were formed, they were ready to be passaged on average every 26.5 ± 3.51 days, in contrast to an average passage time of 5.23 ± 1.01 days with rat neurospheres generated by the typical postmortem dissociation of the SEZ area and grown in the same medium ($p < 0.0001$, using t test analysis). Readiness for passage was determined empirically, at the onset of morphological changes, such as the loss of roundness and the appearance of dark, dense areas. Rather than dissociating the whole-cell culture we chose to harvest neurospheres by lightly agitating the medium, and we subsequently dissociated the spheres with enzymatic treatment and replated them. Milking biopsies resulted on average in 3.17 ± 0.45 passages/harvest, with the ninth passage being the highest observed, while those from saline-injected rats resulted in 1.92 ± 0.76 passages/harvest ($p = 0.038$, t test) (Figure 2I). The passaging capacity of post mortem neurosphere cultures exceeded 12 passages almost in 100% of samples, with a typical expansion (split) capacity of 1–4 per passage. The efficiency of producing positive biopsies and the average passage efficiency did not change with repeated biopsies from the same animal ($n = 9$ rats, 3 biopsies/rat) (data not shown). Notably, the cell-type profile of cells changed over passaging with oligodendroglial lineage cells becoming more abundant in high passages (Figures S1F and S1G). The expansion dynamics of post-milking neursophere colonies ($n = 14$) was calculated in terms of number of neurospheres generated after each passage and was found to be 3.51 ± 2.15, meaning that the number of spheres growing after passaging (data gathered up to the fourth passage) increased on average 3.5 times. Neurospheres of up to passage 4 were tested for multipotency by growing them in medium without growth factors and the generation of all three major CNS cell types (GFAP+ astrocytes, DCX+ immature neurons, and CNPase+ cells of the oligodendroglial lineage) was confirmed (data not shown).

The addition of FGF2 in the release cocktail preserves proliferation in the SEZ

We assessed the effects of milking on the architecture and function of the SEZ at different time points (3–30 dpi) after bilateral injection of a range of low-to-high doses of neuraminidase (100–1,000 mU/injection), as well as with the addition of the integrin-β1-blocking antibody and of the co-injection of FGF2, a pro-mitotic growth factor known to be present in the SEZ (Kerever et al., 2007). Administration of 100 or 250 mU neuraminidase per injection (i.e., per hemisphere) resulted in undetected gross disruption of the ventricular wall (Figure 3D at 7 dpi) and was not used further, while injecting 0.5 and 1 U led to the emergence of multiple sites of ependymal denudation around the site of injection, and to the appearance of neuroblast clusters attached on the ventricular surface (Figures 3E, 3F, S2E,S3A, and S3B). The immunohistochemical analysis of the SEZ in brain sections taken from multiple rostro-caudal levels per animal (and not only at the level of the injection) having undergone milking revealed a significant decrease in the overall density of cells within the stem cell niche at all tested time points and "release" cocktails (Figure 3A). This was not due to the loss of ependymal cells because the volumetric analysis of S100β immunopositive cells in thick brain sections at 7 and 30 dpi, after milking with 0.5 U of neuraminidase, did not reveal any significant overall reduction of the ependyma (Figure S2A). Moreover, the volumetric analysis of astroglial cells revealed no signs of overall significant levels of gliosis (Figure S2C), even though the substitution of denuded ependyma by astrocytes was a typical finding near the injection site (Figure S3; Videos S1 and S2). Additional analyses in thin and thick sections up to 30 dpi revealed that the density of DCX+ neuroblasts remained at control levels irrespective of the release cocktail (Figures 3B and S2B). Neuroblast density was used as a crude output of the function of the SEZ. On the other hand, the density of proliferating cells was significantly decreased compared with saline-injected controls, with the exception of the inclusion of FGF2 in the release cocktail (0.5 μg per injection) (Figure 3C). This suggested a possible detrimental effect on NSPCs, especially to the more mitotic fraction upstream of neuroblasts (Kazanis et al., 2010). To test if this early (at 3 dpi) effect of FGF2

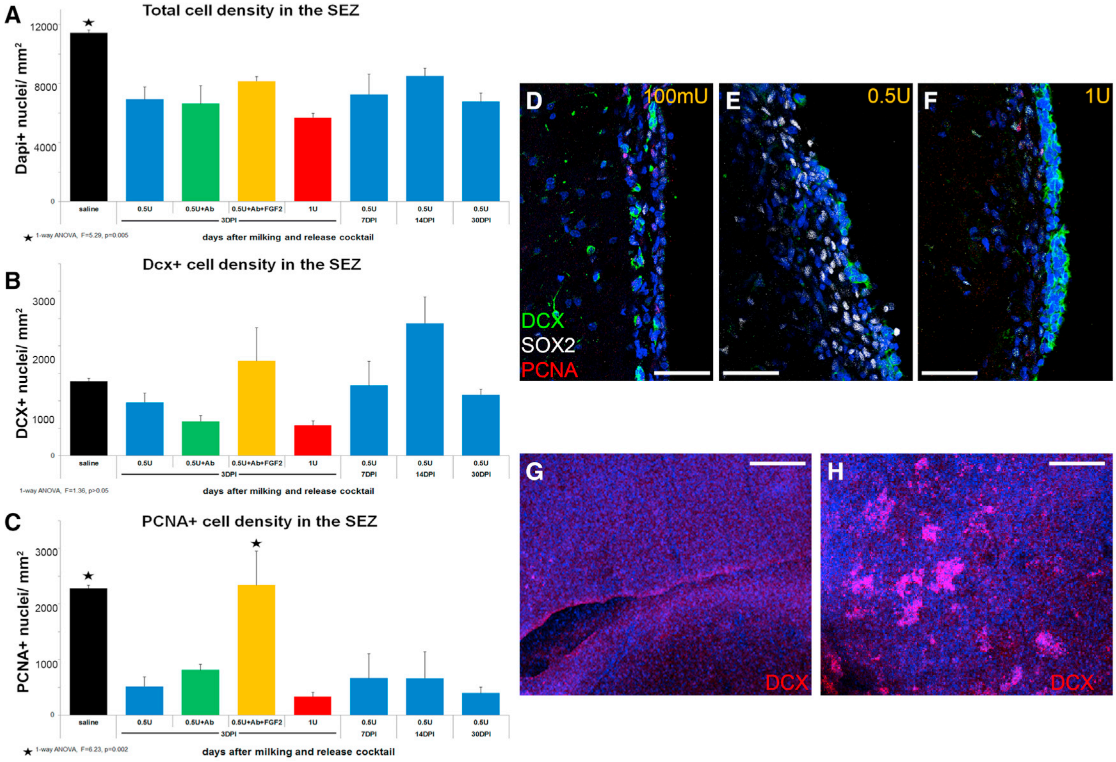

Figure 3. Histological assessment of the effects of milking

(A–C) Graphs showing the average total cell density (A) and that of neuroblasts (B) and proliferating cells (C) within the SEZ at different time points and after the injection of different release cocktails.

(D–F) High-magnification detail of the SEZ after immunostaining for PCNA (to mark proliferating cells), SOX2 (to mark progenitors), and doublecortin (to mark neuroblasts) in brain sections taken from rats injected with release cocktails containing different doses of neuraminidase (100–1,000 mU per injection; 7 dpi).

(G and H) Low-magnification images of the ventricular surface of the SEZ in whole mounts dissected 3 days after the i.c.v. injection of saline (G) or of the release cocktail (500 mU of neuraminidase, 1 μg β1-blocking antibody) (H) and immunostained for doublecortin. This is an "en face" view, as if looking at the ventricular wall from inside the ventricle. Note the emergence of several clusters of neuroblasts, ectopically, at the ventricular surface after milking. Scale bars, 30 μm (D–F) and 0.5 mm (G and H). Error bars: SEM. Statistical analysis in (A–C) as shown under the graphs, n = 4–6 animals per group. See also Figure S2.

in preserving proliferation led to improved histology and function at longer time points, we analyzed the SEZ at 3 and 8 months post-release. We confirmed that the cell density of the SEZ and the density of PCNA+ cells were maintained at overall control levels (Figures 4F and 4G) although, at the level of injection, areas of denuded ependymal were still visible, in many cases complemented by astrogliogenesis (Figure S3; Videos S1 and S2). Due to this last finding, we decided that the inclusion of FGF2 in the release cocktail should be the standard protocol.

Neurogenesis in the SEZ persists irrespective of long-term ependymal loss

The ependyma is an important element of the niche's structure, also providing functional cues to NSPCs (Kazanis and ffrench-Constant, 2012; Lim et al., 2000; Nascimento et al., 2018). We assessed the potential adverse effects of ependymal loss or damage in neurogenesis at a much longer time point (240 dpi of the "+FGF2 release cocktail"), introducing also a higher level of analysis by looking separately at SEZ domains of normal and damaged ependyma. The damage was identified by crude histological assessment or by immunostaining for β-catenin, α-tubulin, or S100β (Shook et al., 2012) (Figures 4A–4E and S4). The overall proliferative activity in the SEZ was not interrupted, with the density of cells undergoing mitosis (PCNA+) remaining at control levels both in domains of intact and damaged ependyma (Figure 4G). Ependymal loss was in many cases replaced by the formation of an astroglial scar at 90 dpi (Figure S3; Videos S1 and S2). Also, similar features, such as areas of ependymal damage/loss accompanied by increased presence of GFAP+ cells and of clusters of neuroblasts on the

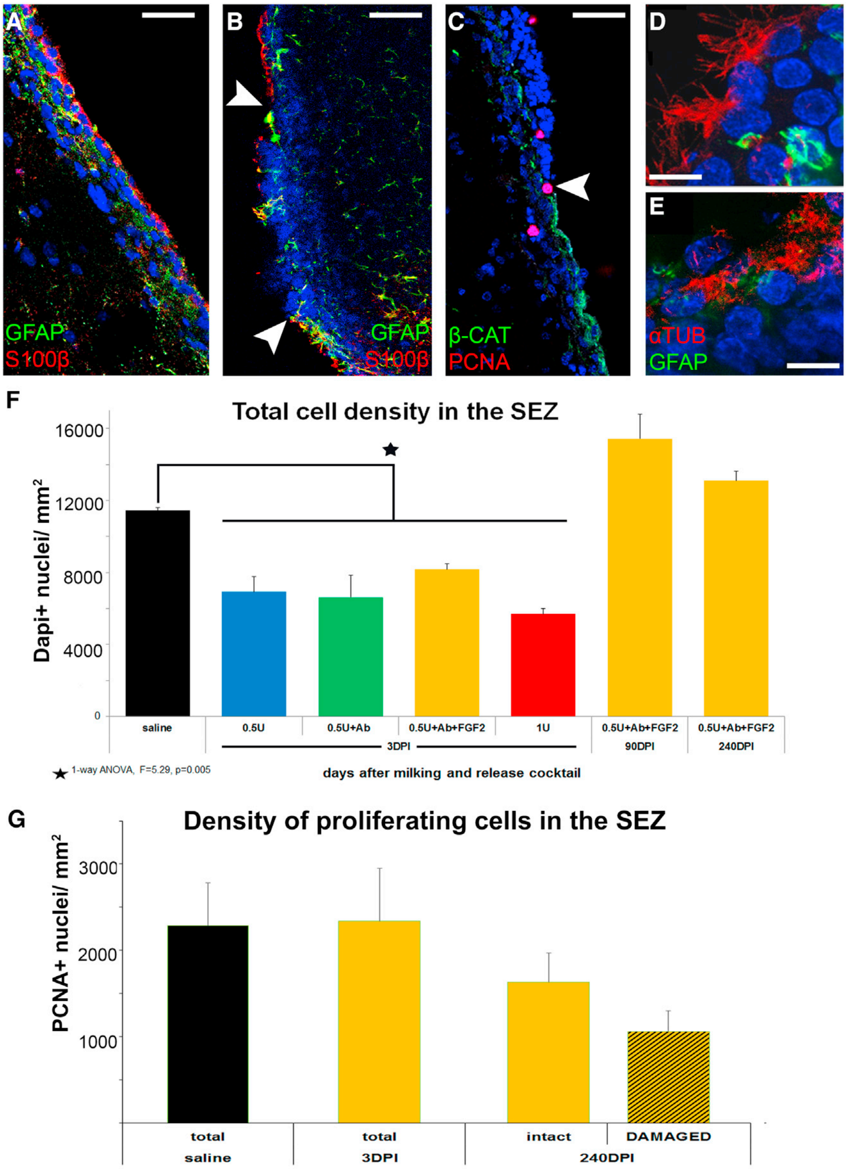

Figure 4. Histological analysis of the SEZ long-term after milking

(A–E) Details of the SEZ 90 dpi of the "+FGF2 release cocktail" after immunostaining for S100β (to mark ependymal cells) with GFAP in (A and B), PCNA (to mark proliferating cells) and β-catenin to mark ependymal cells in (C), or α-tubulin (to observe cilia) and GFAP in (D and E). Note the normal ependymal layer in (A) and the disrupted/damaged ependyma in (B and C). In greater detail, note the normal, multiciliated ependymal cells found in unaffected domains of the SEZ (D) and damaged ones found in affected domains (E).

(F and G) Graphs showing the average total cell density (F) and PCNA+ cells (G) within the saline-injected SEZ and at different time points after the injection of the +FGF2 release cocktail. The PCNA+ cell analysis was performed separately for areas of intact and damaged ependyma at 240 dpi. Scale bars, 5 μm (D and E) and 50 μm (A–C). Error bars: SEM. Statistical analysis: in (F), as shown under the graph, in (G) two-way ANOVA for milking or control and for ependymal damage, followed by post hoc analysis; n = 4–6 animals per group. See also Figures S3 and S4.

ventricular surface, were also observed in animals at 8 months post-release (Figures 1A and S4A–S4D). To explore further what happens directly next to areas of ependymal damage or loss, we quantified the density of SOX2+ NSPCs as well as of SOX2+/GFAP+ double-positive and even triple-positive Ki67+ SOX2+ GFAP+, reactive astrocytes. All these cell populations were found to remain at similar levels, irrespective of ependymal damage (Figures S4E–S4G).

Isolated cells behave as endogenous NSPCs and reflect changes in the SEZ

The choroid plexus has been identified as a key endogenous source of factors that control proliferation of NSPCs in the SEZ (Silva-Vargas et al., 2016). To assess if cells isolated via milking share similar regulatory pathways, they were cultured in choroid plexus-conditioned medium. Indeed, when milking samples were split into two culture conditions, in standard NSPC medium and in choroid plexus-conditioned NSPC medium (n = 5 rats with 2 collections per animal, giving 10 biopsies split in 2 each time), the percentage of positive (having more than 10 cells) biopsies increased to 100% (versus 55.5% when plated in standard NSPC medium in this biopsy cohort, p = 0.023 using paired t test analysis). The immunocytochemical analysis of cell-type profiles revealed a global increase in the numbers of cells grown in the conditioned medium (9.9

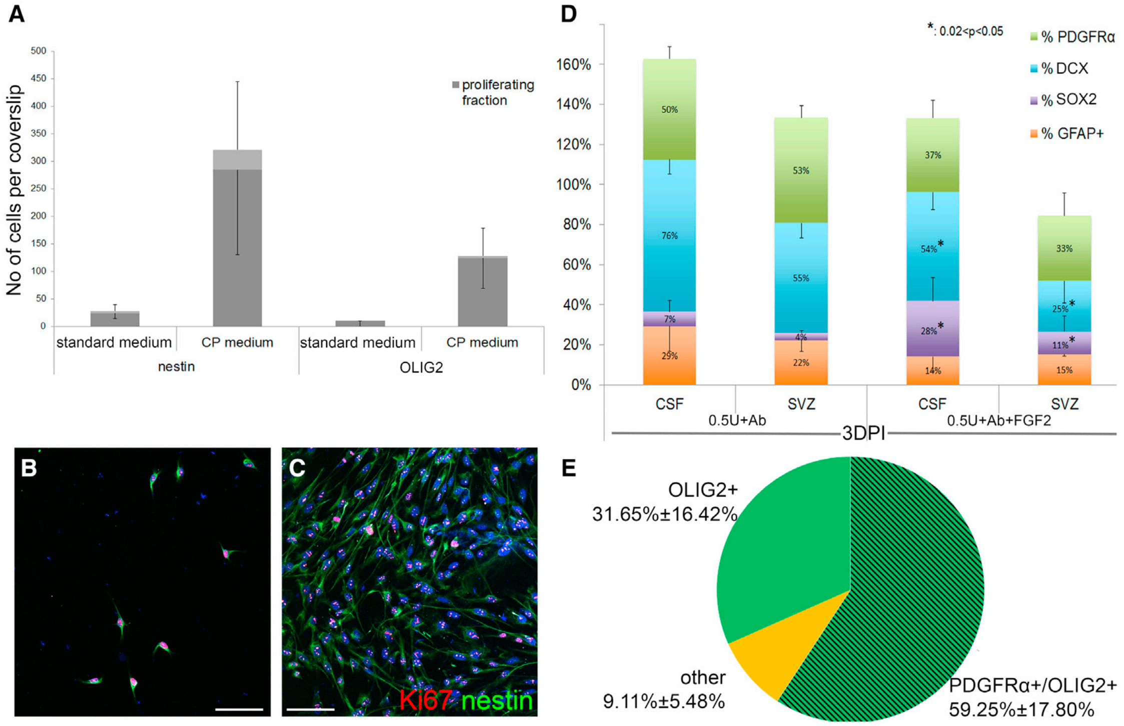

Figure 5. Milking-derived NSPCs reflect the behavior and composition of the endogenous SEZ pool

(A) Graph showing the total number of NESTIN+ and OLIG2+ cells, as well as their proliferating fraction (in dark gray), in milking-derived samples cultured in standard NSPC medium and in medium conditioned with choroid plexus-derived factors (each liquid biopsy was split in two) (n = 3 independent sample experiments).

(B and C) Images taken 7 days after the initial plating of milking-derived cells, grown on PDL-coated glass coverslips in wells of 48-well plates, in standard NSPC medium in (B) and in choroid plexus-conditioned NSPC medium in (C). Cells are immunostained for Ki67 (to mark proliferating cells) and for NESTIN (to mark neural progenitors).

(D) Graph showing the cell-type profile of cells isolated via milking of the SEZ and of the endogenous population of SEZ NSPCs from the same experimental animals. Note the significant increase of the SOX2+ fraction and the significant decrease of the DCX+ fraction after the injection of FGF2 in the lateral ventricles, detected both in the liquid biopsies and in the dissociated SEZs. One-way ANOVA analysis per marker, followed by post hoc analysis, n = 4–6 animals per experimental group.

(E) Graph showing the oligodendroglial cell-type profile of cells isolated after milking of the corpus callosum. Approximately 90% of cells express the key oligodendroglial marker OLIG2, with the majority co-expressing PDGFRα, a marker of OPCs. Scale bar, 100 μm. Error bars: SEM. Statistical analysis per cell-type marker: one-way ANOVA followed by post hoc analysis, n = 4 animals.

times increase of total cells, 11.2 times more NESTIN+ cells, and 10.7 more Ki67+ cells; shown as dark gray portions of the bars in Figure 5A), which was statistically significant for oligodendroglial lineage cells (12.0 times higher number of OLIG2+ cells; p = 0.03 using paired t test analysis) (Figure 5). Notably, though, cells grown in conditioned medium showed decreased self-renewal capacity (number of average passages 2 ± 0.41 in standard medium in this cohort, versus 1.0 in conditioned medium; p = 0.04 using paired t test analysis).

An important potential use of milking the SEZ could be to sample NSPCs as a way of assessing the cell-type profile of the SEZ in live animals; therefore, we investigated if the profile of collected cells accurately reflected changes in the SEZ cell composition. We compared the marker expression of collected cells with that of cells obtained by dissociating the SEZ of the same animals in two experimental groups: after injection of a release cocktail with and without the inclusion of FGF2. We found that the fractions of the different cell types we investigated (Figure 5D) were similar in the SEZ and the respective CSF samples, with the only clear difference being the absence of ASCL1+ cells in the CSF biopsies, in contrast to their 17.23% ± 4.49% pool in the SEZ. The co-injection of FGF2 led to a significant increase in the appearance of SOX2+ cells and to a significant decrease in the appearance of DCX+ neuroblasts both within the SEZ and the liquid biopsies, confirming that changes in the profile of endogenous populations are reflected in isolated cells (Figure 5D).

Isolation of OPCs

Because the ependymal zone is a monolayer also at the ventricular wall underlining the corpus callosum, we assessed if milking can be used to isolate cells of the oligodendroglial lineage from live animals. We performed injections of the release cocktail more caudally, away from the SEZ and next to the hippocampal fimbria (co-ordinates: AP = −1.5 mm, L = ±2.0 mm, D = 3.5 mm) and collected CSF liquid biopsies at 15 dpi. Collected samples were rich in cells expressing the key oligodendroglial transcription factor OLIG2, with the majority exhibiting an OPC profile, as they were immunopositive for both OLIG2 and PDGFRα (Figure 5E).

The human infant ventricular wall shows variable architecture and naturally occurring ependymal damage

In the human brain the pool of NSPCs of the SEZ becomes gradually depleted within the first 18 months after birth (Coletti et al., 2018; Sanai et al., 2011). Nevertheless, similar to the rodent SEZ, NSPCS are separated from the CSF only by ependymal cells (Figures 6E–6G). In cases of rare but devastating neurodegenerative disorders, such as leukodystrophies, that can be diagnosed early during infancy and are incurable, or after perinatal hypoxic/ischemic injury, the SEZ could provide a valuable source of NSPCs. To assess if the human infant SEZ could be milked, we investigated in more detail the architecture and cell-type profile of the ventricular lining (n = 5 samples of perinatal age infants; n = 1 sample from an 18-month-old baby) and we found that ependymal cells express the sialic acid residues (MAA+) that are targeted by neuraminidase (Figures 6A and 6B). We also observed that around birth, the ependyma, which expresses β-catenin (Figure 6C) as has been described in adult rodents (Coletti et al., 2018; Kazanis et al., 2017; Mirzadeh et al., 2008) (Figures 4C and S4A), does not uniquely form a monolayer but also contains thicker domains consisting of numerous ependymal cells (pseudo-layers) (Figure 6A), as well as thick domains formed by GFAP+ cells of radial morphology (Figure 6D), which is similar to the cytoarchitecture of the ventricular wall in newborn mice (Alves et al., 2002). The monolayered architecture was dominant, occupying on average 2.85 times more length than the pseudo-layered ependyma and 6.42 times more length than the radial cell area. Neuroblasts appeared next to both the mono- and pseudo-layered ventricular wall configurations (Figure 6E and 6F), while no neuroblasts were observed at the domains containing radial GFAP+ cells (Figure 6G). By 18 months, the entire lateral ventricle wall was formed by an ependymal monolayer (Figures 6B and 6F); it was void of neuroblasts and showed the well-described, rich in GFAP+ processes, gap zone. Surprisingly, we noticed areas of denuded ependyma in both ages, which in some cases were characterized by gliosis and by the appearance of clusters of neuroblasts on the ventricular surface that were invading the ventricular space, similarly to the post-milking ventricular wall (Figure S5). To investigate if ependymal damage might be also occurring in the rat brain, but under pathological conditions, we performed a histological analysis of the rat SEZ in tissue samples obtained 4 weeks after a hypoxic/ischemic injury induced by 1 h middle cerebral artery occlusion (MCAo) (n = 2). Indeed, we found that, under these conditions, the ependymal layer showed areas of damage, characterized by the flow of SOX2+ NSPCs and DCX+ neuroblasts in the CSF (Figure S6).

DISCUSSION

The ability to isolate postnatal brain NSPCs is of great value both in experimental research and for clinical purposes. Because the human SEZ remains rich in NSPCs for several months after birth (Coletti et al., 2018; Sanai et al., 2011), a method enabling the isolation of SEZ-derived cells would be suitable for providing a source for cell-autologous transplantation strategies. Here, we report a novel method that we have named "milking of the SEZ," which allows the isolation of NSPCs from live rats (Video S3). It is well tolerated by the animals and does not result in a significant overall loss of ependymal cells or of the functionality of the niche. Moreover, if necessary, cell yields could be improved by performing repetitive release cocktail injections per hemisphere, more liquid biopsies, or by increasing the neuraminidase dose injected. Gaining surgical access to the lateral ventricles in infants is plausible, and the performance of CSF biopsies is in many cases standard clinical practice. In addition, we show that human ependymal cells express sialic acid residues that are targeted by neuraminidase and that, in many periventricular areas, the ependymal zone is a monolayer; thus, low-scale denudation is expected to result in the release of NSPCs in the CSF.

Several key data that we have generated suggest that the cells isolated via milking of the SEZ retain the properties of the endogenous pool of NSPCs. Their profile becomes more oligodendrogenic with passages, as has been previously reported for aging SEZ-resident NSPCs (Capilla-Gonzalez et al., 2013; Kazanis et al., 2017). They maintain a slow cell cycle, with an average colony formation time of 26.5 ± 3.51 days. This suggests that the typical colony-forming cell (most likely a NSC, especially in the absence of ASCL1+ transit-amplifying progenitors) becomes activated approximately once every month, in agreement with the slow-cycling behavior of endogenous NSCs (previously characterized as BrdU-retaining cells) (Ferron et al., 2011), but also with the *in vitro* behavior reported after prospective

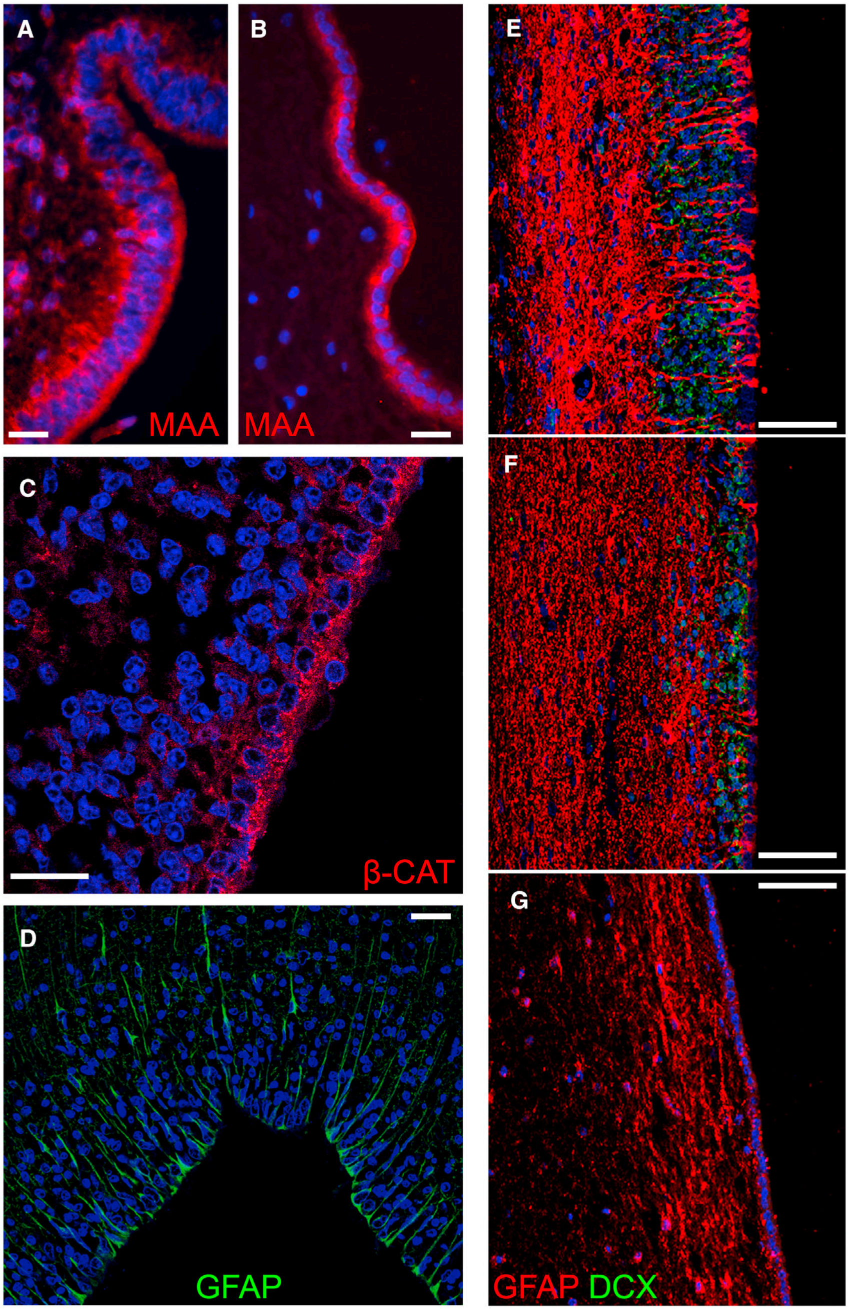

Figure 6. Histological assessment of the human infant and 18-month-old SEZ

(A and B) Immunofluorescence labeling for the biotinylated *Maackia amurensis* lectin (MAA) revealing high presence of sialic acid α(2,3) galactose residues on the ependymal cell layer, both in the newborn brain (A) and in the 18-month-old brain (B). Note the thick (pseudo-layered) ependymal lining of the ventricle in (A).

(C) Image of the SEZ in brain tissue taken from newborn, immunostained for β-catenin, showing the characteristic for the adult rodent brain monolayered ependyma pattern.

(D) Image of the SEZ in brain tissue taken from newborn, immunostained for GFAP, showing the characteristics for the newborn rodent ventricular lining formed by cells of radial glial morphology.

(E–G) Images of the SEZ area in brain tissue taken from newborns (E and F) and an 18-month-old (G) after immunostaining for GFAP and DCX. Note the presence of thicker and thinner neuroblast-rich zones adjacent to the ventricular lining in the newborn SEZ (E and F) and their disappearance in the 18-month-old SEZ (G). Scale bars, 25 μm (A–D) and 50 μm (E–G). See also Figure S6.

identification of different stem cell types (Codega et al., 2014). Notably, the cells isolated via milking of the SEZ showed limited self-renewal potential, in close agreement with results recently produced using elaborate transgenic strategies according to which the majority of activated NSPCs generate clones and subsequently lose their self-renewing potential (Calzolari et al., 2015; Obernier et al., 2018). In our hands, approximately 14% of milking samples showed high passage potential, roughly resembling the 20% fraction of self-renewing NSCs reported by Obernier et al. (2018), while the average 3.17 ± 0.45 passages that we observed is similar to the average colony formation potential of NSPCs estimated by *in vivo* clonal analyses (Calzolari et al., 2015). The rest of the samples, and especially the 42% of “0” passage collections, contain neural progenitors with limited self-renewal properties or other cells (such as ependymal cells) with zero self-renewal capacity. Finally, when the profile of the SEZ pool of NSPCs

was exogenously induced to change by i.c.v. injection of FGF2, the composition of cells isolated via milking was changed in a similar pattern.

Overall, our data indicate that this protocol provides a way to sample repeatedly, and in the long-term, the endogenous population of NSPCs of the SEZ with high fidelity, possibly because it does not involve the "aggressive" dissociation of the tissue that is necessary when isolating NSPCs with the standard post mortem protocols, which generate cultures with significantly higher mitotic activity. Work on muscle satellite (stem) cells has shown that the (standard) culture of cells in a high proliferative activity mode comes with the caveat of missing key quiescence properties characteristic of the endogenous stem cells (Peault et al., 2007). Further to this evidence, experimental work on isolated intestinal stem cells has revealed that preserving the quiescence properties of stem cells in cultures is crucial in order to allow them to exhibit their full cell generation range, as described by *in vivo* analyses (Basak et al., 2017). Our data revealed that culture medium conditioned with choroid plexus factors induced the proliferation of isolated cells, as expected based on previous reports (Silva-Vargas et al., 2016), but at the same time significantly decreased their self-renewal capacity. In a recent paper, platelet-derived growth factor D has been identified as a component of the CSF that acts via PDGFRβ to enhance quiescence of NSCs (Delgado et al., 2021). By transferring the targeted area of the ventricular wall more caudally and away from the SEZ niche, we were able to isolate cells with typical OPC marker profile. Furthermore, the ability to perform successive liquid biopsies in significant depth of time (histological data clearly reveal that NSPCs cluster in direct contact with the CSF even at 8 months post-release) in the same animal will allow for the first time to investigate the biology of NSPCs of the SEZ stem cell niche and of OPCs in longitudinal experimental studies. Such approaches are indispensable in order to assess the sequence of events within one individual animal and to enhance the implementation of the principles of the "3Rs" (replacement, reduction, and refinement) aiming at performing more humane animal research.

Milking is based on the targeted damage of the ependyma, a cellular element of the NSC niche known to be important for providing a structural barrier toward the ventricular space (as we confirm here), but also as a regulator of NSPC function (Kazanis and ffrench-Constant, 2012; Lim et al., 2000; Nascimento et al., 2018; Wu et al., 2020). Our data confirm and extend recent observations in GemC1 knockout mice in which ependyma fails to form. In these mice, astrocytes seem to take over from ependymal cells, but neurogenesis persists (they survive for about 1 month after birth due to other health problems) (Lalioti et al., 2019). In our experiments the animals remain healthy; therefore, our investigation of the effects of ependymal ablation was extended to several months. The detailed analysis of the niche in areas of normal and damaged ependyma revealed a focal gliotic reaction, but without overall increased presence of astroglial cells, as well as normal levels of mitotic activity and preservation of neurogenesis. This supports the notion that reactive astrocytes might be providing a supportive microenvironment for NSPCs, while short-term infusion of β1- and α6-integrin-blocking antibodies has been shown to enhance proliferation in the SEZ (Kazanis et al., 2010; Shen et al., 2008). On the other hand, observations in perinatal human tissue revealing ependymal damage accompanied both by astrogliosis and by clusters of neuroblasts invading the ventricle, combined with the documented exhaustion of neurogenic activity at 18 months (Coletti et al., 2018; Sanai et al., 2011), warrant further investigation regarding the possible contribution of early ependymal damage to NSPC loss in the human niche. By milking the SEZ we now provide an experimental tool that allows the complementary investigation of human and rodent tissue in order to address this scenario.

Our data revealed that 32.1% of liquid biopsies in rats without the prior injection of a release cocktail were positive in cells; although, with a significantly lower self-renewing potential (1.92 ± 0.76, never exceeding fourth passage), possibly suggesting that they did not contain stem cells but only progenitors. Nevertheless, this finding suggests that low numbers of NSPCs could be isolated by repeated liquid biopsies in saline-injected animals. Furthermore, when we assessed the SEZ structure in rats that had been subjected to 1 h MCAo, a model of hypoxic/ischemic injury, we also identified features similar to those induced via milking, primarily the emergence of ependymal damage and of clusters of neuroblasts flowing into the ventricular space. All the above suggest that, in certain experimental and clinical conditions, NSPCs could be collected from the CSF without the necessity to induce their release.

EXPERIMENTAL PROCEDURES

Animal welfare

Adult male and female Sprague-Dawley, Wistar, or Long-Evans rats with body weights between 170 and 250 g were used (Charles River Laboratories, or inbred in the animal facilities of the Universities of Cambridge and Patras). Animal breeding, maintenance, and experimental procedures were conducted in accordance with the UK Animals (Scientific Procedures) Act 1986, authorized by the Home Office and with the Presidential Decree 56/2013 of the Hellenic Republic, scrutinized by the Animal Welfare and Ethical Review Bodies of the Universities of Cambridge and Patras. The milking procedure was well tolerated by animals, with no lethality connected to the manipulation, although the release cocktail injection

surgery resulted in significantly higher levels of body weight loss immediately after the surgery when compared with saline injections, albeit below the point of concern (weight changes 2 dpi: saline [n = 5] 0.85% ± 0.84%; "0.5 U neuraminidase + blocking antibody" [n = 7]: −1.91% ± 0.57% [p = 0.004 compared with saline]; "+FGF2" [n = 8]: −3.72% ± 1.93% [p < 0.0001 compared with saline], using one-way ANOVA followed by Scheffe's post hoc). We did not identify any gross histological changes that could explain this difference, but the administration of analgesia was necessary to keep levels of weight loss at a minimum. At 50 dpi, animals had gained similar body weight irrespective of milking (+FGF2:10.62% ± 1.30%; saline: 9.30% ± 1.30%). Performing up to three successive liquid biopsies (typically, one before milking and the rest afterward, at intervals of at least 7 days) did not result in any detectable effects on the well-being of the animals (weight gain, mobility, behavior), or the gross histology of periventricular areas (data not shown).

Milking of the SEZ and of the corpus callosum

Detailed protocol provided in the supplemental experimental procedures.

The release cocktail (containing different combinations of: *Clostridium perfringens* neuraminidase [100 mU–1 U], 1 μg integrin-β1-blocking antibody, 0.5 μg FGF2), or sterile saline, was bilaterally injected at a rate of 1 μL/min at the following co-ordinates (relatively to bregma): AP axis of −0.3 mm, L axis of ±2.0 mm, D of 3.5 mm to milk the SEZ, or, AP axis of −1.5 mm, L axis of ±2.0 mm, D of 3.5 mm to milk the corpus callosum. For the infusion of the release cocktail via osmotic mini-pumps, a cannula (BIK-II, Alzet) was fixed on the skull (1 mm lateral to bregma) connected to a subcutaneously implanted miniosmotic pump (1003D, Alzet) filled with neuraminidase solution to deliver intracerebroventricularly 2 U/day over 3 days (6 U total) at an infusion rate of 1 μL/h.

At different time points after surgery, a CSF liquid biopsy using the stereotaxic frame (the head positioned at a downward 40° angle) and a 1-mL insulin-like syringe. Once CSF appeared, further suction was applied to enable CSF flow at a rate of 40 μL/min, producing a blood-free sample of up to 120 μL. The liquid biopsy was mixed with 400 μL of NSPC medium (DMEM [Thermo Fisher Scientific], B27 supplement [2%, v/v] [Thermo Fisher Scientific], 20 ng/mL FGF2 [Peprotech], and 20 ng/mL EGF [Peprotech]) and was kept at 4°C until further use.

MCAo

The MCAo procedure has been published elsewhere (Augestad et al., 2017). In brief, right MCAo was performed under anesthesia (n = 3) using the intraluminal filament technique. The animals remained anesthetized for 60 min when the monofilament was withdrawn to allow for reperfusion and recovery; brain tissue was collected 4 weeks post MCAo.

Tissue processing and immunohistochemistry

Details provided in the supplemental experimental procedures.

Animals were culled by transcardial infusion of 4% paraformaldehyde and tissue was cut using either a cryostat or a vibratome. Immunofluorescence stainings were performed using standard protocols. Images were acquired using Leica SP5 and SP8 confocal microscopes and were processed using ImageJ (NIH, USA) and LasX (Leica) software.

Human tissue

Human tissue samples were generously provided by the UK Brain Banks Network (all derived from the Oxford Brain Bank) and from the Greek Brain Bank, a member of the Brain Net Europe (BNE) after securing proper consent. We worked with paraffin-embedded tissue, mounted on glass slides, derived from four male and one female infants who died at birth, all with no neuropathological findings (postmortem delays in fixation ranging from 24 to 96 h). One sample was from an 18-month-old male, who died due to anorectal abscess and other congenital abnormalities of the intestine, with no neuropathological findings (postmortem delay to fixation 48 h). All sections included parts of the SEZ, as advised by the Bank's neuropathologists.

CSF and SEZ samples for cell-type profiling

Collected CSF samples were transferred into tubes containing NSPC culture media with 50 mM HEPES and were kept on ice until further handling (addition of 4 mL of medium, followed by centrifugation at 800 × *g* for 5 min). Cells were plated into chamber slides (Thermo Fisher Scientific), or 96-well plates, coated with poly-D-lysine and laminin (10 μg/mL, Sigma, L2020). Specifically for immunostaining, cells were plated in Greiner CELLSTAR 96-well plates. Cells were fixed in ice-cold 2% paraformaldehyde for 10 min. For SEZ profiling, the brain and then the SEZ were dissected and put in 500 μL of DMEM. The SEZ tissue was gently triturated using progressively smaller pipettes. Subsequently it was digested in 200 μL papain solution (1 mL medium, 40 μL papain, 10 μL DNAse) for 45 min. Digestion was stopped with 5 mL NSPC culture medium (+10% FBS) and the cell suspension was spun at 1,000 × *g* (5 min) and resuspended in 7 mL of medium, from which 2 × 200 μL were transferred to two wells of a chamber slide. Cells were then immunostained using standard procedures (see supplemental experimental procedures). For OPC collections, CSF samples were mixed with OPC proliferation medium (DMEM [GlutaMAX, 14.5g/L D-glucose, -pyruvate)] supplemented with 1% N_2, 1 mM biotin, 0.05% BSA-FFa [Sigma-Aldrich], 60 mg/mL cysteine [Sigma-Aldrich], 1% P/S, 10 ng/mL FGF2 [Peprotech], and 10 ng/mL PDGF-AA [Peprotech]) in which cells were plated until being fixed at 24 h, ahead of immunostaining.

Long-term cell cultures and choroid plexus-conditioned medium assays

To allow cells to settle, only half of the medium was replaced every 3 days. Neurospheres were harvested via gentle manipulation with a pipette, while the initial population of adherent single cells remained intact. Neurospheres or adherent cell colonies were passaged with accutase (STEMCELL Technologies). For choroid plexus-conditioned medium, rat pups were culled at weaning age (postnatal days 21–25) and their choroid plexi were dissected out. Each plexus was put in one well of a 96-well plate with 200 μL of NSPC culture medium. The medium was collected after 48 h, cleared of debris with spinning, and kept at 4°C. Fresh medium (200 μL) was put in the same well for another 48 h and subsequently the plexus was discarded (400 μL of total conditioned medium produced per choroid plexus dissection). CSF samples

used for experiments in control and conditioned media were split in two and plated in wells of 48-well plates on poly-D-lysine-coated 9-mm glass coverslips.

3D reconstructions and volumetric measurements

Image stacks of the tissue sections were acquired on a Leica TCS SP8 confocal microscope using the ×40 lens and with a z step spacing of 0.8 μm. The software used for the 3D imaging and subsequent calculations was Bitplane Imaris (7.4.2). Firstly, a rectangular surface (region of interest [ROI]) was selected along the dorsoventral axis, which was selected to be approximately 30 μm in width and contained the first few cells of the ependyma. Each fluorescence channel within the ROI was transformed into a 3D object using a "surfaces area detail level" value of 0.5 μm and the threshold was set to "absolute intensity." Manual adjustments were made where deemed necessary in order to match the 3D object appearance to the fluorescent signal as closely as possible. Dapi, S100β, DCX, and GFAP were measured as a total volume of their respective 3D object voxels. The final measurements were expressed as a percentage of their corresponding ROI.

Cell counts and statistics

For the investigation of the effects of milking at various time points (as shown in Figures 2A–2C), images were acquired with the ×40 objective lens from at least two sections from four rostrocaudal levels (+2.0, +1.0, +0.5, and −0.5 mm in respect to bregma) of the forebrain. In each section, three optical fields were imaged: two in the dorsal horn of the SEZ and one in the middle SEZ. For the dorsal horn, the area of the tissue analyzed was measured (either in ImageJ or LasX software), while for the middle SEZ, cells were counted at a depth of 50 μm from the ventricular surface (Kazanis and ffrench-Constant, 2012) and the area was calculated by multiplying with the respective length of the ventricular wall. Cell densities are given as "number of cells per mm^2" and cell counts were performed using the counter plugin of LasX or the respective tool of the Bio-format importer plugin of ImageJ. Co-expression of various molecules was assessed by marking positive cells independently for each immunostaining and subsequently stacking all markings together. For analysis at the level of the release cocktail injection (as shown in Figures 2I and 2J), the areas of ependymal damage were identified and at least two optical fields were taken using the ×63 objective lens per domain. Statistical analyses were performed using Microsoft Office Excel or GraphPad Prism software. When comparing different post-lesion time points, one-way ANOVA was used, followed by post hoc tests. When comparing post-release and saline-injected rat CSF biopsies Student's t-test analysis was used. Statistical significance was always set to $p = 0.05$.

SUPPLEMENTAL INFORMATION

Supplemental information can be found online at https://doi.org/10.1016/j.stemcr.2021.08.015.

AUTHOR CONTRIBUTIONS

F.McC. designed the data acquisition, and analyzed and interpreted the data. C.D. designed the data acquisition, analyzed and interpreted the data, and contributed to drafting the manuscript. C.K. helped in the acquisition of data, carried out analysis of the data, and contributed to drafting the manuscript. D.D., A.A., D.-K.M., E.A., P.K., M.K., S.O., M.P., and R.-T.R. assisted in the acquisition and analysis of the data. R.J.M.F. analyzed and interpreted the data, and contributed to drafting the manuscript. I.K. conceived and designed the data acquisition, analyzed and interpreted the data, and contributed to drafting the manuscript.

CONFLICTS OF INTEREST

The authors declare no competing interests.

ACKNOWLEDGMENTS

This work was supported by an Action Medical Research (UK) grant (GN2291) to R.J.M.F. and I.K. It was also funded in whole, or in part, by the Wellcome Trust (203151/Z/16/Z) and the UKRI Medical Research Council (MC_PC_17230). For the purpose of open access, the author has applied a CC BY public copyright licence to any Author Accepted Manuscript version arising from this submission. Rat tissue with MCAo injury was generously provided by Dr. Ioanna Sandvig (Norwegian University of Science and Technology). We are grateful to Professors Anastasia Konstantinidou and Maria Panayotacopoulou (Medical School, University of Athens) for supplying human samples.

Received: December 8, 2019
Revised: August 23, 2021
Accepted: August 24, 2021
Published: September 23, 2021

REFERENCES

Alves, J.A., Barone, P., Engelender, S., Froes, M.M., and Menezes, J.R. (2002). Initial stages of radial glia astrocytic transformation in the early postnatal anterior subventricular zone. J. Neurobiol. *52*, 251–265.

Augestad, I.L., Nyman, A.K.G., Costa, A.I., Barnett, S.C., Sandvig, A., Håberg, A.K., and Sandvig, I. (2017). Effects of Neural Stem Cell and Olfactory Ensheathing Cell Co-transplants on Tissue Remodelling After Transient Focal Cerebral Ischemia in the Adult Rat. Neurochem Res. *42*, 1599–1609. https://doi.org/10.1007/s11064-016-2098-3.

Basak, O., Beumer, J., Wiebrands, K., Seno, H., van Oudenaarden, A., and Clevers, H. (2017). Induced quiescence of Lgr5+ stem cells in intestinal organoids enables differentiation of hormone-producing enteroendocrine cells. Cell Stem Cell *20*, 177–190 e174.

Calzolari, F., Michel, J., Baumgart, E.V., Theis, F., Gotz, M., and Ninkovic, J. (2015). Fast clonal expansion and limited neural stem cell self-renewal in the adult subependymal zone. Nat. Neurosci. *18*, 490.

Capilla-Gonzalez, V., Cebrian-Silla, A., Guerrero-Cazares, H., Garcia-Verdugo, J.M., and Quinones-Hinojosa, A. (2013). The generation of oligodendroglial cells is preserved in the rostral migratory stream during aging. Front. Cell. Neurosci. *7*, 147.

Codega, P., Silva-Vargas, V., Paul, A., Maldonado-Soto, A.R., Deleo, A.M., Pastrana, E., and Doetsch, F. (2014). Prospective

identification and purification of quiescent adult neural stem cells from their in vivo niche. Neuron 82, 545–559. https://doi.org/10.1016/j.neuron.2014.02.039.

Coletti, A.M., Singh, D., Kumar, S., Shafin, T.N., Briody, P.J., Babbitt, B.F., Pan, D., Norton, E.S., Brown, E.C., Kahle, K.T., et al. (2018). Characterization of the ventricular-subventricular stem cell niche during human brain development. Development 145, dev170100.

Culver, J.C., Vadakkan, T.J., and Dickinson, M.E. (2013). A specialized microvascular domain in the mouse neural stem cell niche. PLoS One 8, e53546.

Del Carmen Gomez-Roldan, M., Perez-Martin, M., Capilla-Gonzalez, V., Cifuentes, M., Perez, J., Garcia-Verdugo, J.M., and Fernandez-Llebrez, P. (2008). Neuroblast proliferation on the surface of the adult rat striatal wall after focal ependymal loss by intracerebroventricular injection of neuraminidase. J. Comp. Neurol. 507, 1571–1587.

Delgado, A.C., Maldonado-Soto, A.R., Silva-Vargas, V., Mizrak, D., von Känel, T., Tan, K.R., Paul, A., Madar, A., Cuervo, H., Kitajewski, J., Lin, C.S., and Doetsch, F. (2021). Release of stem cells from quiescence reveals gliogenic domains in the adult mouse brain. Science 372, 1205–1209. https://doi.org/10.1126/science.abg8467.

Doetsch, F., Caille, I., Lim, D.A., Garcia-Verdugo, J.M., and Alvarez-Buylla, A. (1999). Subventricular zone astrocytes are neural stem cells in the adult mammalian brain. Cell 97, 703–716.

Doetsch, F., Petreanu, L., Caille, I., Garcia-Verdugo, J.M., and Alvarez-Buylla, A. (2002). EGF converts transit-amplifying neurogenic precursors in the adult brain into multipotent stem cells. Neuron 36, 1021–1034.

Etxeberria, A., Mangin, J.M., Aguirre, A., and Gallo, V. (2010). Adult-born SVZ progenitors receive transient synapses during remyelination in corpus callosum. Nat. Neurosci. 13, 287–289.

Ferron, S.R., Charalambous, M., Radford, E., McEwen, K., Wildner, H., Hind, E., Morante-Redolat, J.M., Laborda, J., Guillemot, F., Bauer, S.R., et al. (2011). Postnatal loss of Dlk1 imprinting in stem cells and niche astrocytes regulates neurogenesis. Nature 475, 381–385.

Kazanis, I., Evans, K.A., Andreopoulou, E., Dimitriou, C., Koutsakis, C., Karadottir, R.T., and Franklin, R.J.M. (2017). Subependymal zone-derived oligodendroblasts respond to focal demyelination but fail to generate myelin in young and aged mice. Stem Cell Reports 8, 685–700.

Kazanis, I., and ffrench-Constant, C. (2012). The number of stem cells in the subependymal zone of the adult rodent brain is correlated with the number of ependymal cells and not with the volume of the niche. Stem Cells Dev. 21, 1090–1096.

Kazanis, I., Lathia, J.D., Vadakkan, T.J., Raborn, E., Wan, R., Mughal, M.R., Eckley, D.M., Sasaki, T., Patton, B., Mattson, M.P., et al. (2010). Quiescence and activation of stem and precursor cell populations in the subependymal zone of the mammalian brain are associated with distinct cellular and extracellular matrix signals. J. Neurosci. 30, 9771–9781.

Kerever, A., Schnack, J., Vellinga, D., Ichikawa, N., Moon, C., Arikawa-Hirasawa, E., Efird, J.T., and Mercier, F. (2007). Novel extracellular matrix structures in the neural stem cell niche capture the neurogenic factor fibroblast growth factor 2 from the extracellular milieu. Stem Cells 25, 2146–2157.

Lalioti, M.E., Kaplani, K., Lokka, G., Georgomanolis, T., Kyrousi, C., Dong, W., Dunbar, A., Parlapani, E., Damianidou, E., Spassky, N., et al. (2019). GemC1 is a critical switch for neural stem cell generation in the postnatal brain. Glia 67, 2360–2373.

Lim, D.A., Tramontin, A.D., Trevejo, J.M., Herrera, D.G., Garcia-Verdugo, J.M., and Alvarez-Buylla, A. (2000). Noggin antagonizes BMP signaling to create a niche for adult neurogenesis. Neuron 28, 713–726.

Loulier, K., Lathia, J.D., Marthiens, V., Relucio, J., Mughal, M.R., Tang, S.C., Coksaygan, T., Hall, P.E., Chigurupati, S., Patton, B., et al. (2009). beta1 integrin maintains integrity of the embryonic neocortical stem cell niche. PLoS Biol. 7, e1000176.

Luo, J., Shook, B.A., Daniels, S.B., and Conover, J.C. (2008). Subventricular zone-mediated ependyma repair in the adult mammalian brain. J. Neurosci. 28, 3804–3813.

Mercier, F., Kitasako, J.T., and Hatton, G.I. (2002). Anatomy of the brain neurogenic zones revisited: fractones and the fibroblast/macrophage network. J. Comp. Neurol. 451, 170–188.

Mirzadeh, Z., Merkle, F.T., Soriano-Navarro, M., Garcia-Verdugo, J.M., and Alvarez-Buylla, A. (2008). Neural stem cells confer unique pinwheel architecture to the ventricular surface in neurogenic regions of the adult brain. Cell Stem Cell 3, 265–278.

Mouret, A., Lepousez, G., Gras, J., Gabellec, M.M., and Lledo, P.M. (2009). Turnover of newborn olfactory bulb neurons optimizes olfaction. J. Neurosci. 29, 12302–12314.

Nascimento, M.A., Sorokin, L., and Coelho-Sampaio, T. (2018). Fractone bulbs derive from ependymal cells and their laminin composition influence the stem cell niche in the subventricular zone. J. Neurosci. 38, 3880–3889.

Obernier, K., and Alvarez-Buylla, A. (2019). Neural stem cells: origin, heterogeneity and regulation in the adult mammalian brain. Development 146, dev156059.

Obernier, K., Cebrian-Silla, A., Thomson, M., Parraguez, J.I., Anderson, R., Guinto, C., Rodas Rodriguez, J., Garcia-Verdugo, J.M., and Alvarez-Buylla, A. (2018). Adult neurogenesis is sustained by symmetric self-renewal and differentiation. Cell Stem Cell 22, 221–234 e228.

Pastrana, E., Cheng, L.C., and Doetsch, F. (2009). Simultaneous prospective purification of adult subventricular zone neural stem cells and their progeny. Proc. Natl. Acad. Sci. U S A 106, 6387–6392.

Peault, B., Rudnicki, M., Torrente, Y., Cossu, G., Tremblay, J.P., Partridge, T., Gussoni, E., Kunkel, L.M., and Huard, J. (2007). Stem and progenitor cells in skeletal muscle development, maintenance, and therapy. Mol. Ther. 15, 867–877.

Sanai, N., Nguyen, T., Ihrie, R.A., Mirzadeh, Z., Tsai, H.H., Wong, M., Gupta, N., Berger, M.S., Huang, E., Garcia-Verdugo, J.M., et al. (2011). Corridors of migrating neurons in the human brain and their decline during infancy. Nature 478, 382–386.

Shen, Q., Wang, Y., Kokovay, E., Lin, G., Chuang, S.M., Goderie, S.K., Roysam, B., and Temple, S. (2008). Adult SVZ stem cells lie

in a vascular niche: a quantitative analysis of niche cell-cell interactions. Cell Stem Cell *3*, 289–300.

Shi, Y., Inoue, H., Wu, J.C., and Yamanaka, S. (2017). Induced pluripotent stem cell technology: a decade of progress. Nat. Rev. Drug Discov. *16*, 115–130.

Shook, B.A., Manz, D.H., Peters, J.J., Kang, S., and Conover, J.C. (2012). Spatiotemporal changes to the subventricular zone stem cell pool through aging. J. Neurosci. *32*, 6947–6956.

Silva-Vargas, V., Maldonado-Soto, A.R., Mizrak, D., Codega, P., and Doetsch, F. (2016). Age-dependent niche signals from the choroid plexus regulate adult neural stem cells. Cell Stem Cell *19*, 643–652.

Theunissen, T.W., and Jaenisch, R. (2014). Molecular control of induced pluripotency. Cell Stem Cell *14*, 720–734.

Wu, J., Tian, W.J., Liu, Y., Wang, H.J., Zheng, J., Wang, X., Pan, H., Li, J., Luo, J., Yang, X., et al. (2020). Ependyma-expressed CCN1 restricts the size of the neural stem cell pool in the adult ventricular-subventricular zone. EMBO J. *39*, e101679.

Stem Cell Reports, Volume *16*

Supplemental Information

Isolation of neural stem and oligodendrocyte progenitor cells from the brain of live rats

Freyja McClenahan, Christina Dimitriou, Christos Koutsakis, Dimitrios Dimitrakopoulos, Asterios Arampatzis, Paraskevi Kakouri, Michaela Kourla, Sofia Oikonomou, Evangelia Andreopoulou, Melina Patsonis, Danai-Kassandra Meri, Rana-Tahir Rasool, Robin JM. Franklin, and Ilias Kazanis

Supplemental Figures and Legends; Legends to Supplemental Movies

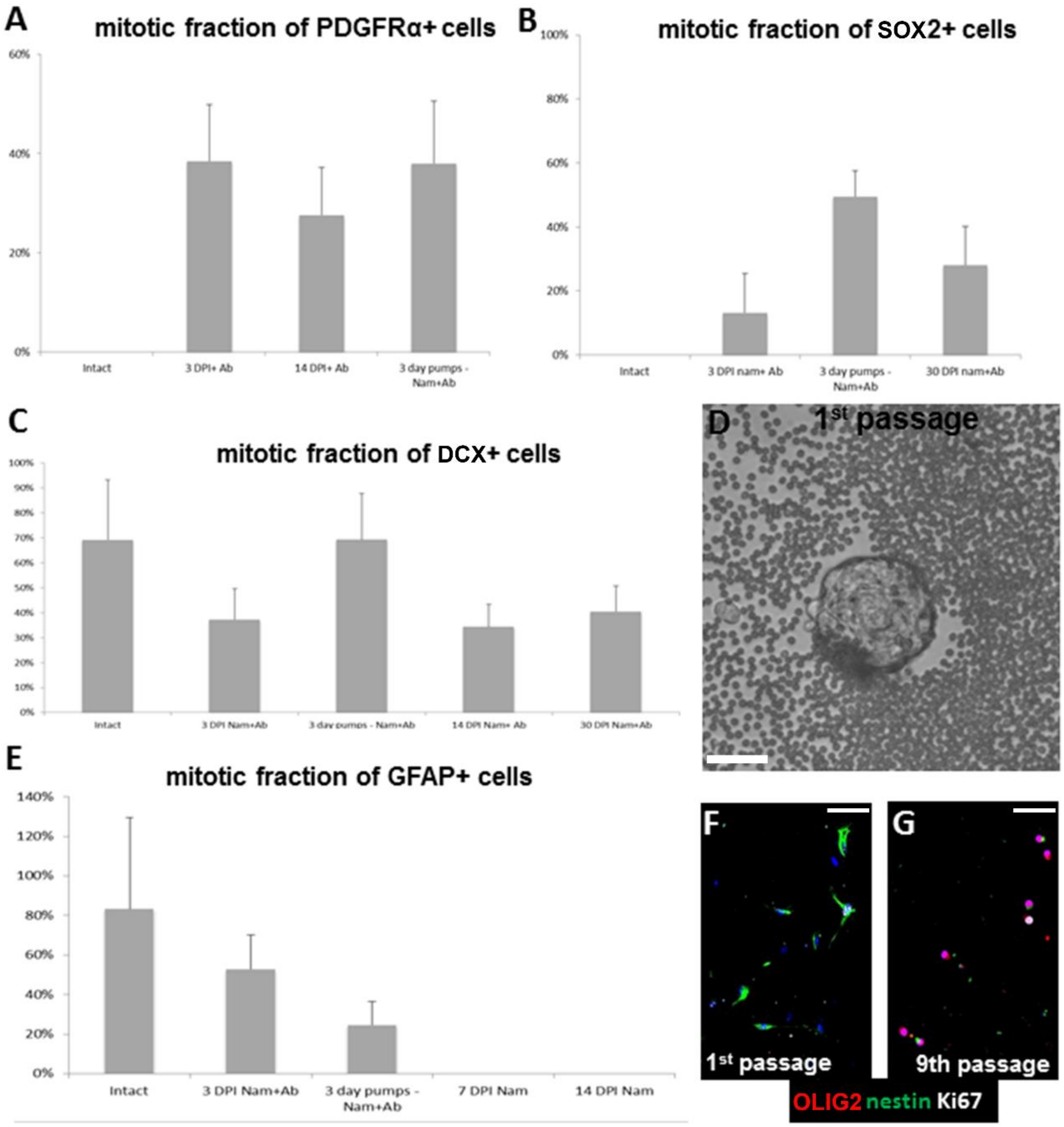

Suppl. Figure 1 (Related to Fig2)

Mitotic profile of cells in liquid biopsies

(A-C,E) Graphs showing the percentage of PCNA/PDGFRα (in A), PCNA/SOX2 (in B), PCNA/DCX (in C) and PCNA/GFAP (in E) double-positive cells collected per liquid biopsy of CSF at different time-points after the "basic release cocktail" (500mU neuraminidase, 1μg β1-integrin blocking Ab) injection as well as after 3 days of infusion via minipump. [error bars: SEM, statistical analysis using one-way ANOVA; n=3-6 animals per experimental group]. (D) Characteristic brightfield image of a primary neurosphere growing in the presence of erythrocytes. (F-G) Images of cells immunostained for OLIG2, nestin and Ki67, showing increased presence of oligodendroglial lineage cells in high passages. [scale bars: 50μm in D, 25μm in F,G]

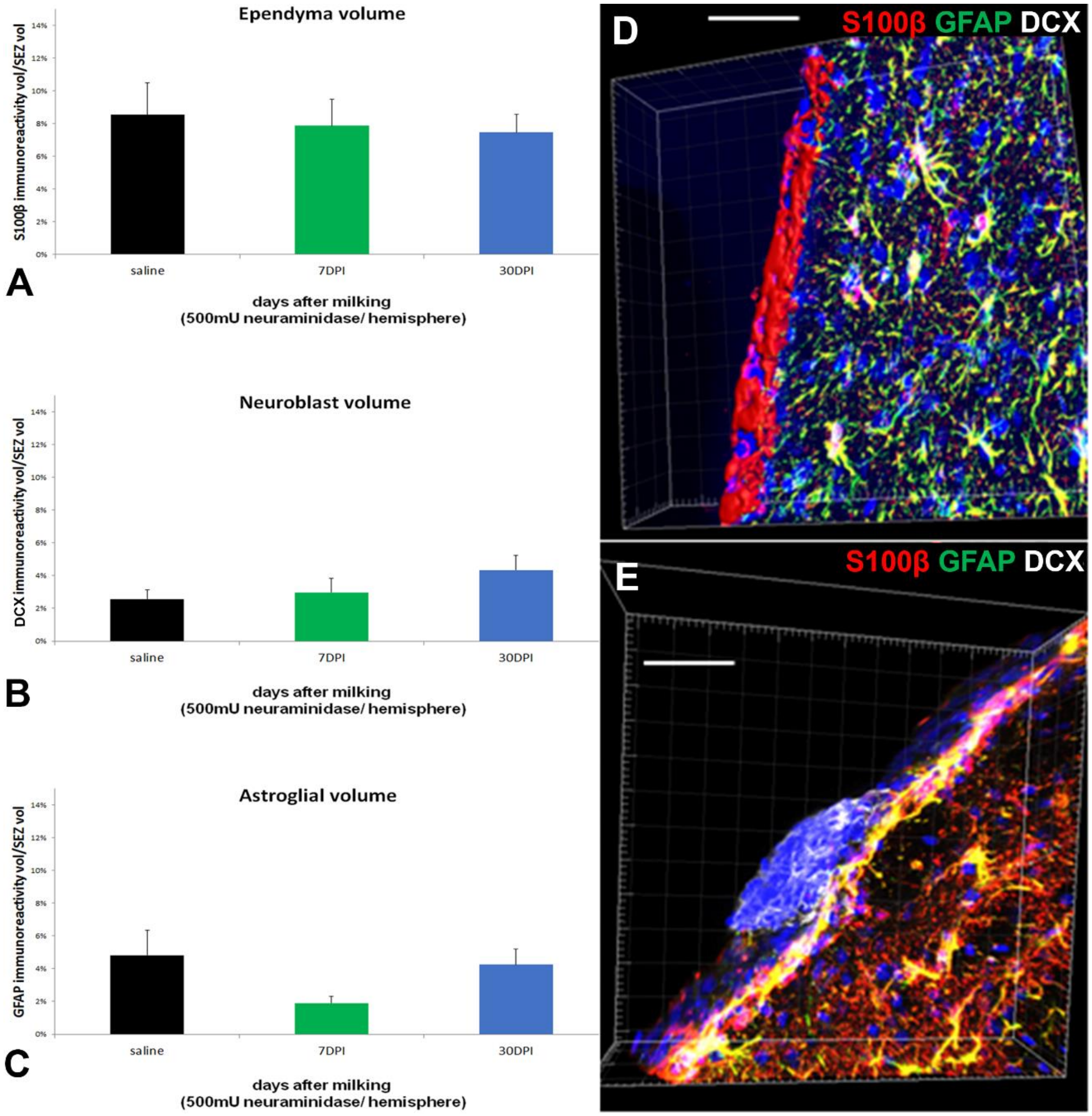

Suppl. Figure 2 (Related to Fig4 and Fig5)

Volumetric analysis of the post-milking SEZ

(A,C) Graphs showing the volume of the ependyma, of neuroblasts and of astrocytes (as volume fraction of S100β, DCX and GFAP immunopositive cell-somata and processes, respectively, per total SEZ volume) at different time-points after the injection of the release cocktail that contained 0.5U neuraminidase per injection (D,E). Representative Imaris-produced images of the saline-injected (in D) and of the 7DPI (in E) SEZ area after immunostaining for S100β, GFAP and DCX. Note a cluster of neuroblasts at the ventricular surface after milking of the SEZ in (E). [error bars: SEM; scale bar:150μm; one-way ANOVA, n=3 animals per experimental group]

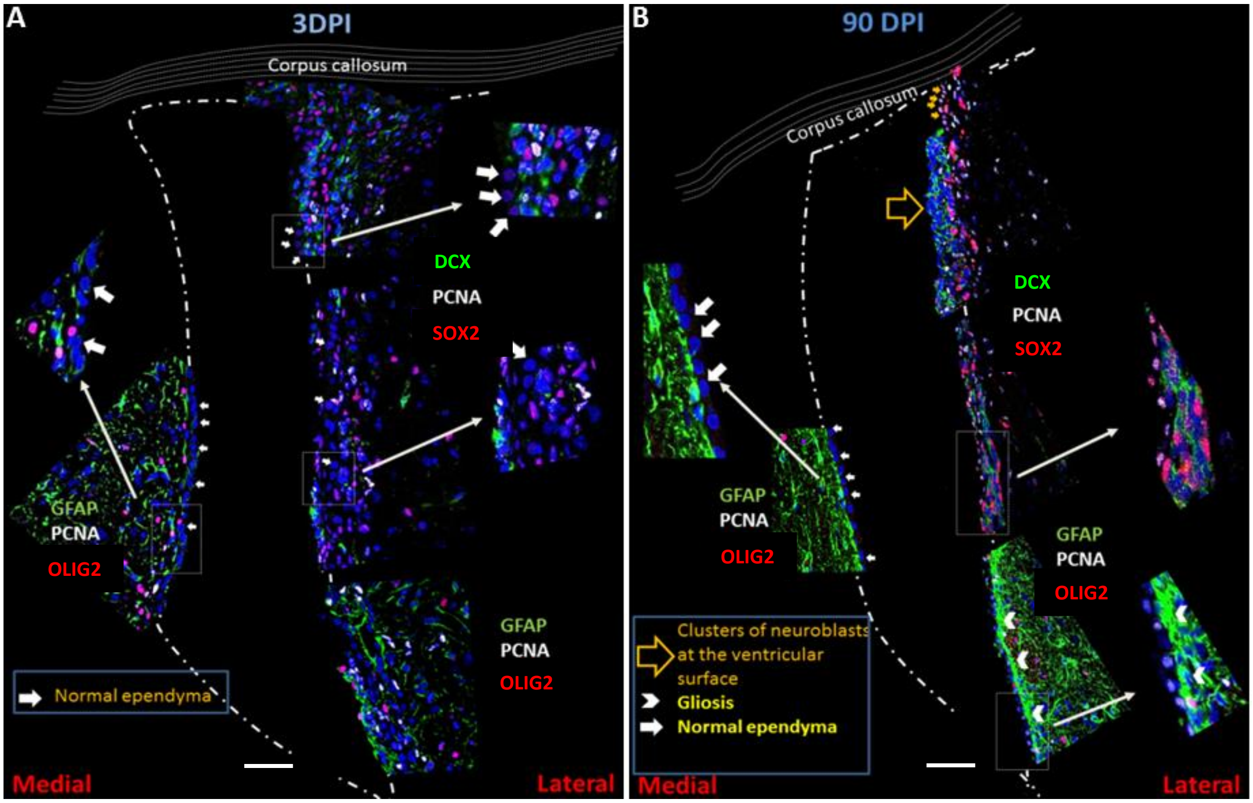

Suppl. Figure 3 (Related to Fig4)

Histological analysis of the post-milking SEZ
(A,B) Collage of images taken from the SEZs of rats at 3 days (in A) and 90 days (in B) after the injection of the "0.5U neuraminidase + blocking Ab" release cocktail. The collages are constructed with images taken from different sections of the same animals, after immunostaining for DCX/PCNA/SOX2 and GFAP/PCNA/OLIG2. Note areas of normal ependyma (indicated by white arrows), especially at the medial (non-neurogenic) wall of the lateral ventricles, as well as the existence of clusters of neuroblasts on the ventricular surface at 3 months post-injection. Also, note the emergence of areas of gliosis 3 months post-milking [scale bar: 50µm].

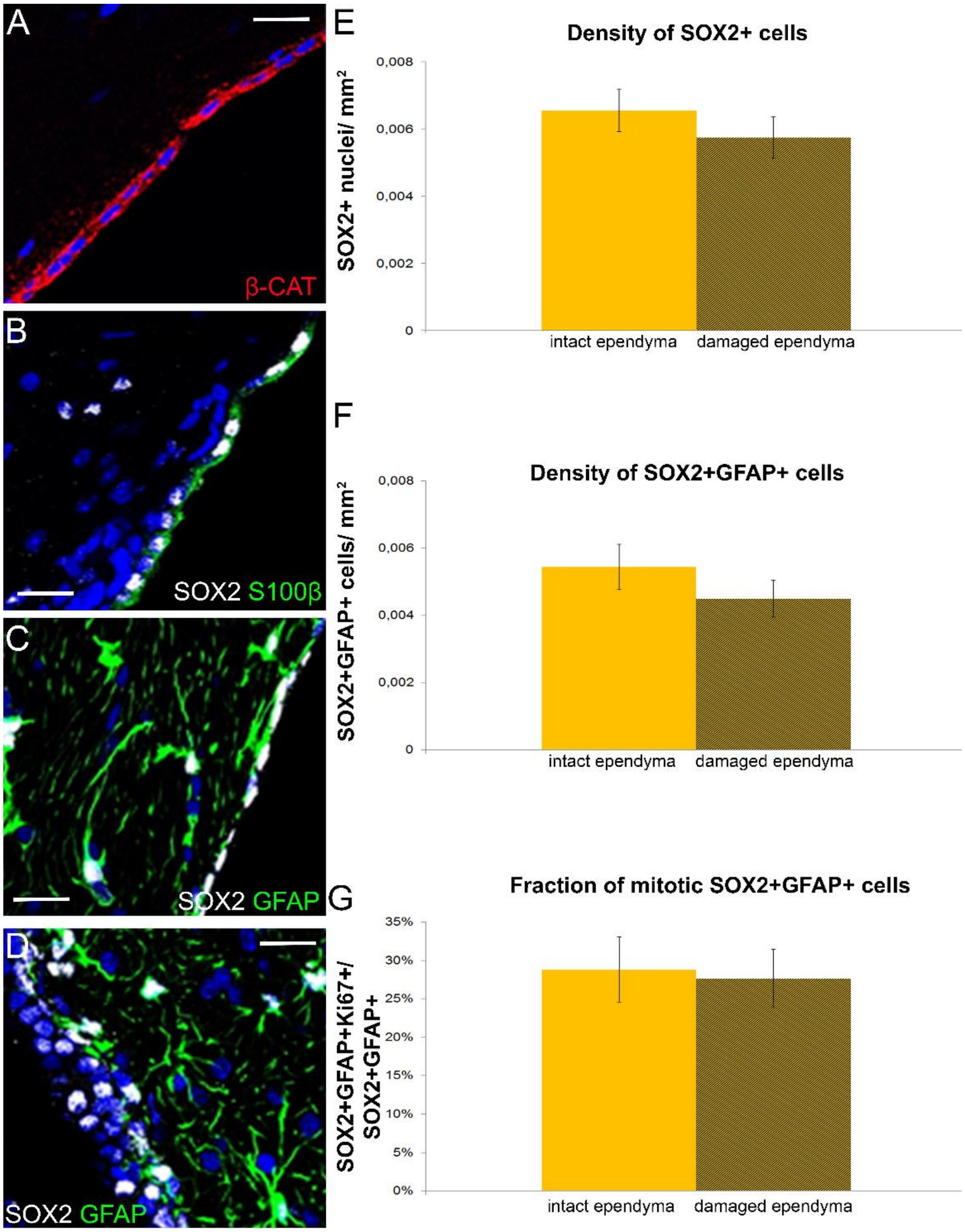

Suppl. Figure 4 (Related to Fig5)

Histological analysis of the SEZ in respect to ependymal damage at 8 months post-injection

(A-D) Details of the SEZ 8 months (240DPI) after the injection of the "+FGF2 release cocktail" and after immunostaining for β-catenin (in red, in A, to mark ependymal cells), SOX2 (in white in B-D), S100β (in green, in B, to mark ependymal cells) and GFAP (in green, in C-D). Images were taken from the same animal, with those in A-C, depicting SEZ domains with intact ependymal (note the smooth ependymal monolayer) and that in D depicting a detail from an area of damaged (note the lack of a continuous SOX2+ monolayer). (F-G) Graphs showing the average percentages of total SOX2+ ependymal and NSPC, of SOX2/GFAP double positive, activated astrocytes and of mitotic, activated astrocytes, in areas adjacent to intact and damaged ependyma. [scale bar: 20μm; error bars: SEMs, statistical analysis paired t-test; n=4 animals]

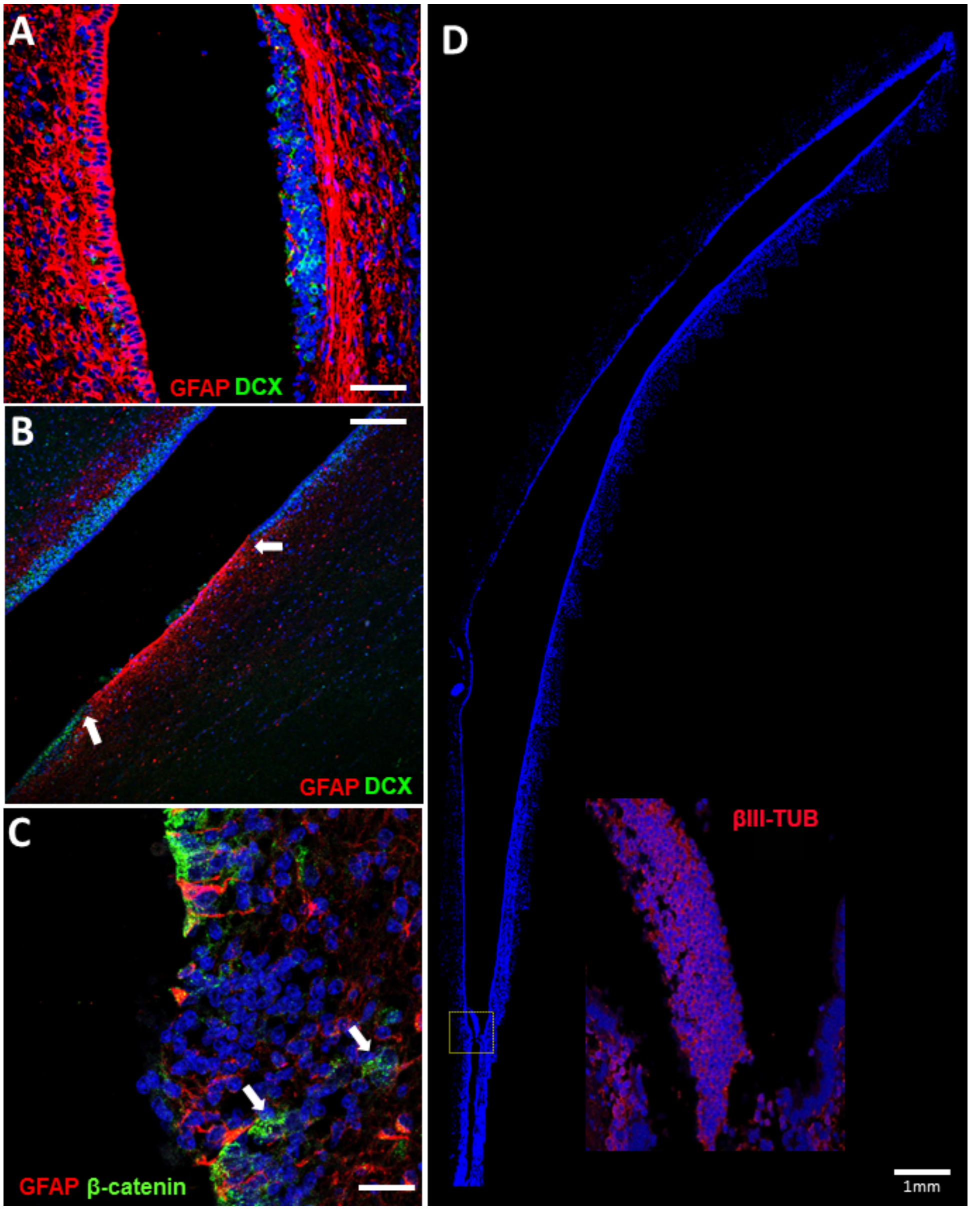

Suppl. Figure 5 (Related to Fig6)

Detection of ependymal disruption, gliosis and neuroblast clustering in the human infant SEZ
Images of the SEZ in tissue taken from newborn infants and immunostained for several markers reveal areas of gliosis and overlaying clusters of neuroblasts (in A and B; gliotic scar indicated by arrows in B) and of groups of neuroblasts flowing in the CSF (D; the boxed area is shown in magnification in the inset). Immunostaining for β-catenin helps visualize the disruption of the ependymal layer (in C) with some ependymal cells surviving deeper in the tissue (indicated with arrows in C). [scale bars: 50µm in A,C; Image D is a collage of multiple photos of Dapi staining]

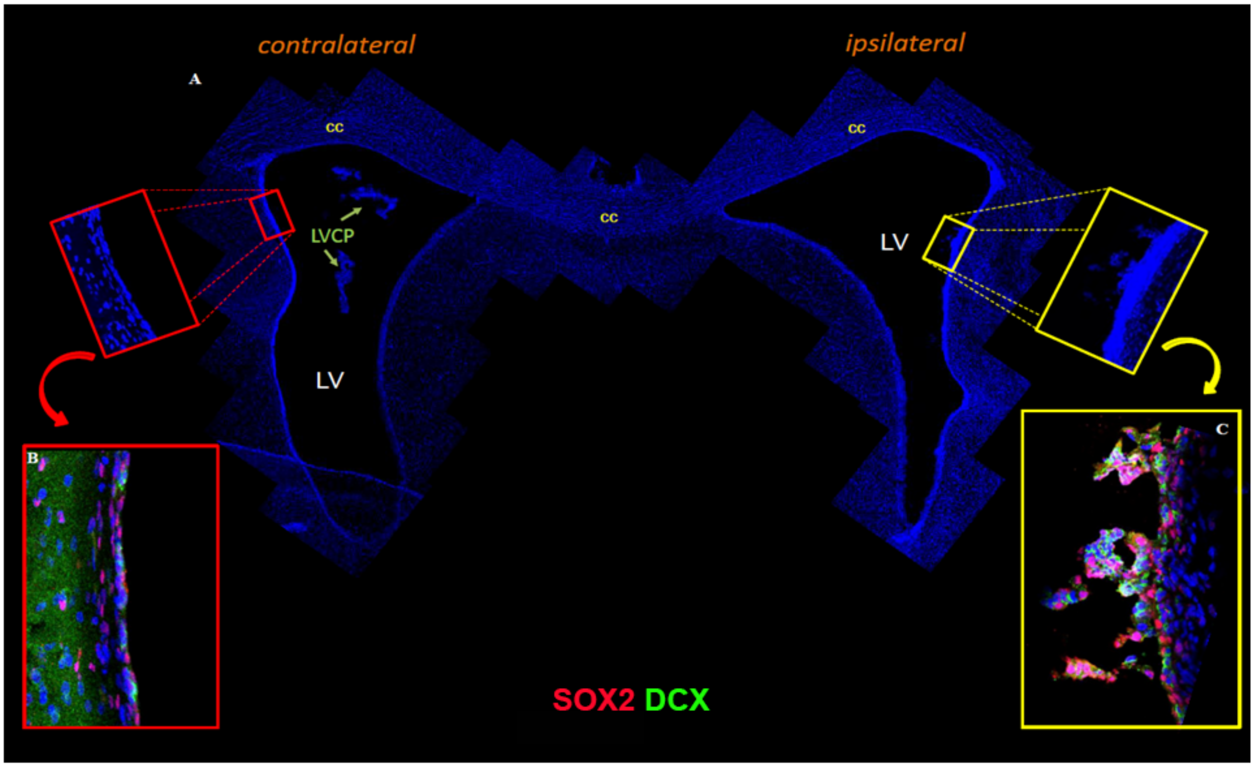

Suppl. Figure 6 (Related to Fig6)

Detection of ependymal disruption and neuroblast clustering in the SEZ post hypoxia/ ischaemia

Collage of images showing the lateral ventricles of a rat 4 weeks after hypoxic/ischaemic injury (60min of Middle Cerebral Artery Occlusion/ MCAO) and after immunostaining for DCX and SOX2. Ependymal disruption accompanied by the flow of SOX2+/DCX+ cells in the ventricular space is observed at the affected hemisphere (at the right).[LV: Lateral Ventricle; LVCP: Lateral Ventricle Choroid Plexus; cc: corpus callosum

Suppl. Movie 1 (Related to Fig1,3)
Detail of the intact SEZ ventricular surface
Movie generated by confocal-microscopy-derived stack of images, taken from rat tissue after immunostaining for GFAP. Note the GFAP-negative lining of the ventricular wall (made of ependymal cells).

Suppl. Movie 2 (Related to Fig1,3)
Detail of the post-milking SEZ ventricular surface
Movie generated by confocal-microscopy-derived stack of images, taken from rat tissue 90 days post-milking, after immunostaining for GFAP. Note the existence of only sporadic GFAP-negative cells on the ventricular wall (surviving ependymal cells) and the gliotic scar that is forming at the ventricular wall.

Suppl. Movie 3 (Related to all Figures)
Summary animation of the "milking" method.

Supplementary Experimental Procedures (Related to Experimental Procedures)

Tissue processing and immunohistochemistry

Animals were culled by transcardial infusion of 4% paraformaldehyde (PFA), tissue was post-fixed overnight in 2% PFA (at 4°C) and was cryo-preserved in 30% sucrose (in Phosphate Buffer Saline/PBS) for 48h at 4°C before freezing at -50°C (for cryostat) or storage in anti-freezing solution at -20°C (for vibratome). Sections were cut with a Leica cryostat (12 mm thick), or a Leica vibratome (70µm) and were processed for immunohistochemistry using the primary antibodies listed below. Whole mount preparations of the SEZ were performed as previously described[2].

Immunofluorescence stainings were performed using standard protocols, that included incubation with blocking buffer (3%BSA, 0.1% Triton x-100 from Sigma, UK, in PBS) and, depending on the requirement, antigen retrieval (15min boiling in 10mM citrate buffer, pH=6.0). Human tissue was provided in the form of thin paraffin sections; thus, they underwent a deparaffinization/rehydration process before further manipulation. Primary antibody incubation (in blocking buffer) was performed for one or two overnights at 4°C and incubation with secondary antibodies (in PBS with Dapi for nuclear staining) for 2h at room temperature. Slides were covered with coverslips mounted with mowiol. Images were acquired using Leica SP5 and SP6 confocal microscopes and were processed using ImageJ (NIH, USA) and LasX (Leica) software.

Antibodies and Lectin

Rabbit anti-OLIG2 (1/200, Millipore, AB9610) and rabbit anti-PDGFRα (1/200, Abcam, 1/200, ab51875) were used to identify OPCs. Rabbit, goat, chicken or mouse anti-GFAP (Dako, 1/500, Z0334; Abcam, 1/500, ab53554; Abcam, 1/500, ab4674; Sigma, 1/500, G3893) and rabbit anti-DoublecortinCX (Abcam, 1/500, ab18723) or mouse anti-TUBULIN βIII (Sigma, 1/500, T8578) were used for astrocytes and neuroblasts respectively. To assess progenitor identityidentity, we used a monoclonal anti-NESTIN (1/200, Abcam, ab6142) and a goat anti-SOX2 antibody (Santa Cruz, 1/200, sc-17320). Proliferating cells were identified using a mouse anti-PCNA (Abcam, 1/500, Ab29), a rabbit anti-Ki67 (Abcam, 1/500, ab15580), or a rabbit anti-PH3 (Abcam, 1/500, ab80612) antibody. Ependymal cells were identified using a mouse anti-S100β (Sigma, 1/200, S2532), a rabbit anti-acetylated α-TUBULIN (Sigma, 1/500, T6793) and a rabbit anti-β-CATENIN (Abcam, 1/500, ab16051). Transit amplifying progenitors were marked with an anti-Ascl1 (MASH-1) mouse monoclonal antibody (1/100, Clone 24B72D11.1, (RUO) BD Bioscience, 556604). The

appropriate secondary antibodies were used, purchased from Thermo Fisher (Molecular probes, Alexa conjugated 488, 568 and 647).

Neuraminidase-targeted sialic acid residues (Sialic acid α(2,3) galactose) were identified by binding with Maackia amurensis lectin (MAA, BA-7801-2, EY Laboratories Inc.) that was biotin-conjugated and was subsequently visualized using Streptavidin conjugated with Alexa 594 (Thermo Fisher).

Detailed milcking protocol

A. “Release cocktail” composition (prepared at the day of the procedure and kept on ice. Quantities given per 2µl to be injected i.cv in each lateral ventricle):

1. 500mU Neuraminidase from Clostridium perfringens (*Clostridium welchii*) (#N2876,Sigma-Aldrich) [kept as a 1U/µl stock diluted in sterile water at -20°C]**[0.5µl]**
2. 1µg β1-integrin blocking antibody (#555002, BD Biosciences) [purified NA/LE Hamster Anti-Rat CD29 Clone Ha2/5, 1mg/ml, kept at 4°C]**[1µl]**
3. 0.5µg Fibroblast Growth Factor (#100-18B, Peprotech)[Recombinant Human FGF-basic (154 a.a.), kept as a 1µg/µl stock diluted in sterile water at -20°C]**[0.5µl]**

Note: prepare an additional 1µl per intended injection

B. Injection of “release cocktail”:

Note: surgical procedures were performed under general anaesthesia induced by inhaled isofluorane (2.5% for induction and 2% for maintenance), but can be successfully performed under injectable anaesthesia (e.g. ketamine/ xylazine). On average the procedure lasted for 30min and analgesia (Vetergesic; Buprenorphine 0.3mg/ml as buprenorphine hydrochloride 0.324 mg/ml) was given i.p. upon induction of anaesthesia.

4. Standard procedures for mounting the animal on the stereotaxic frame are followed. An incision of the head’s skin along the middle line is performed, followed by meticulous clearing of the skull, identification of bregma and the drilling of 1mm burr holes, using a dental drill, at the following co-ordinates (from bregma): Anterioposterior axis +0.5mm, Lateral axis ±1.4mm).
5. A loaded 10µl Hamilton syringe is fixed on the stereotaxic device and the needle (preferably blunt or conical edge) is brought in contact with the dura and then inserted 3.5mm deep.

6. The "release cocktail" is infused at a rate of 1μl/min and then the needle is left for another 2min before being retrieved. The procedure is repeated at the other hemisphere.
7. Incisions are sutured and the animal is transferred to the post-operation monitoring area.

C. CSF liquid biopsy:

Note: surgical procedures were performed under general anaesthesia induced by inhaled isofluorane (2.5% for induction and 2% for maintenance), but can be successfully performed under injectable anaesthesia (e.g. ketamine/ xylazine). On average the procedure lasted for 10min; analgesia was given at the induction of anaesthesia.

8. The animal is mounted on the stereotaxic frame using only the ear bars; thus, allowing free forward/backward rotation of the head.
9. The head is stabilized at a downward 40° angle so that a good extension of the back of the neck can be achieved; the fur is shaved and the skin is cleaned.
10. A depressible surface with the appearance of a rhomb between the occipital protuberance and the spine of the atlas can be identified using the tip of a finger (Consiglio and Lucion, 2000; Pegg et al., 2010).
11. A 1ml insulin-like syringe is fixed on the stereotaxic frame and a 27G needle is attached on it (syringes with compact (non-detachable) needles are preferable as suction is more efficient and there is less dead-volume). The needle is lowered almost at contact point to the skin at the center of the identified rhomb. After the syringe is lowered further through the skin layers, small suction is applied to create negative pressure. The syringe is lowered at very small steps until CSF starts to appear due to the negative pressure.
12. The syringe is stabilized at this point and further suction is applied to enable more CSF flow. The needle can be lowered or elevated slightly. The suction of CSF, at a rate of 40μl/ min, will produce a clear from blood sample of up to 120μl. The syringe must be removed slowly and the rat should be supported by an i.p. injection of 1ml normal serum.
13. The liquid biopsy is mixed with 400μl of NSPC medium [DMEM (Thermo Fisher), B27 supplement (2% v.v) (Thermo Fisher), 20ng/ml FGF2 (Peprotech) and 20ng/ml EGF (Peprotech)] and is kept at 4°C until further use. (*Note: all liquid biopsies were processed the latest after 4h)*
14. The animal is transferred to the post-operation monitoring area.

References

Consiglio, A.R., and Lucion, A.B. (2000). Technique for collecting cerebrospinal fluid in the cisterna magna of non-anesthetized rats. Brain Res Brain Res Protoc *5*, 109-114.

Pegg, C.C., He, C., Stroink, A.R., Kattner, K.A., and Wang, C.X. (2010). Technique for collection of cerebrospinal fluid from the cisterna magna in rat. J Neurosci Methods *187*, 8-12.
